# Kinetics and mechanisms in lipopolysaccharide-induced neuroinflammation: a framework for phytochemicals screening

**DOI:** 10.1007/s11033-026-12817-4

**Published:** 2026-10-08

**Authors:** Muhammad Mazhar Munir, Xian Zhou, Dennis Chang

**Affiliations:** 1 NICM Health Research Institute, Western Sydney University, Westmead, NSW 2145 Australia; 2 Department of Pharmacology and Toxicology, Cholistan University of Veterinary and Animal Sciences, Bahawalpur, 63100 Pakistan

**Keywords:** Neuroinflammation, Lipopolysaccharide, Phytochemical, Microglia, TLR4, NF-κB

## Abstract

Neuroinflammation is an innate immune response of the central nervous system in which microglia, astrocytes and neuronal stress pathways regulate the production of cytokines, inflammatory enzymes and redox mediators. Among experimental paradigms, lipopolysaccharide (LPS) provides a stimulus for probing inflammatory signalling and for evaluation of phytochemicals. However, heterogeneity in LPS provenance, chemotype, purification grade, dose, route, exposure duration and endpoint timing compromises cross-study comparability, mechanistic attribution and translational interpretation. This review synthesises evidence from in vitro and in vivo LPS-induced neuroinflammation models used for phytochemical screening and proposes a kinetics- and mechanism-informed framework for experimental design. In vitro, murine microglial systems predominate, particularly BV-2, N9 and primary microglia, with complementary use of astroglia, neuronal and human myeloid or microglia-like platforms. LPS concentrations range from 100 ng/mL to 1 µg/mL, with signalling activation occurring within minutes to hours and inflammatory phenotypes typically assessed at 24–48 h. Frequently used pharmacodynamic readouts include tumour necrosis factor-α, interleukin-1β, interleukin-6, inducible nitric oxide synthase/nitric oxide, cyclooxygenase-2/prostaglandin E2, reactive oxygen species, glial activation markers and viability indices, combined with mechanistic interrogation of NF-κB, MAPK, PI3K–Akt, Nrf2–HO-1 and NLRP3-associated pathways. In vivo, repeated intraperitoneal LPS administration predominates in rodent models, whereas intracerebroventricular and zebrafish models provide complementary spatial and kinetic insights. Overall, LPS challenge should be interpreted as a controlled inflammatory trigger rather than a disease-equivalent surrogate. Rigorous LPS reporting, closer alignment of endpoints with inflammatory kinetics, and multi-endpoint validation across models should strengthen mechanistic inference and support translational evaluation of phytochemical leads.

**Graphical abstract:**

Kinetics and mechanism informed framework for phytochemical screening in LPS-induced neuroinflammation. Created in BioRender. Zhou, P. (2026) https://BioRender.com/8jznpa5.

**Figure:**
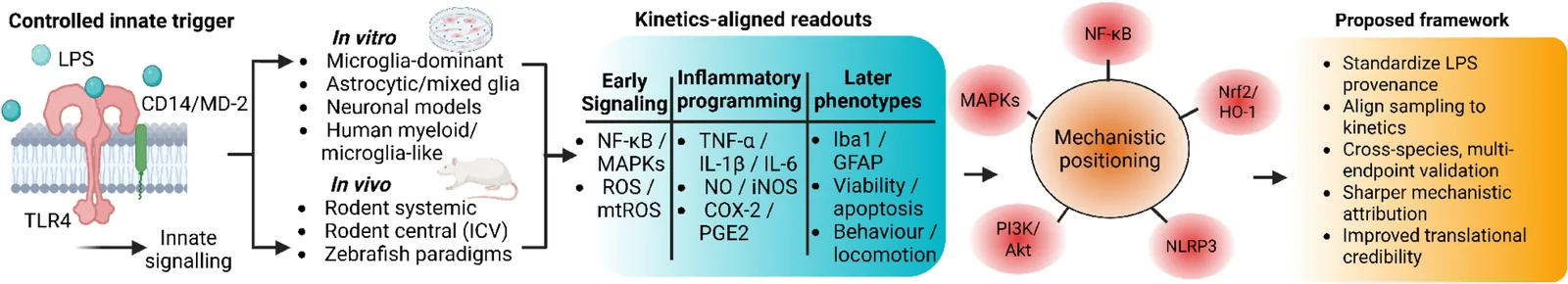

**Supplementary Information:**

The online version contains supplementary material available at https://doi.org/10.1007/s11033-026-12817-4.

## Introduction

Neuroinflammation is a coordinated response within the central nervous system (CNS), characterized by the inducible production of cytokines, chemokines, and inflammatory enzymes. Although the blood–brain barrier (BBB) confers a degree of immune privilege, the CNS is actively surveyed by resident immunocompetent cells, principally microglia and astrocytes, which collectively regulate inflammatory set points and outcomes [1, 2]. Under physiological conditions, these glial populations contribute to the maintenance of tissue homeostasis; upon sensing danger signals, they act as key innate immune effectors that amplify or resolve neuroinflammatory responses.

LPS, a Gram-negative bacterial endotoxin, activates Toll-like receptor 4 (TLR4) in cooperation with CD14/MD-2 and is widely used to induce a controllable, stimulus-defined innate immune response in glial systems. TLR4 engagement triggers canonical inflammatory signalling that culminates in quantifiable outputs including tumour necrosis factor-α (TNF-α), interleukin-1β (IL-1β), interleukin-6 (IL-6), and induction of inducible nitric oxide synthase (iNOS)/nitric oxide (NO) and reactive oxygen species (ROS)/oxidative stress [3, 4]. Although acute activation can be adaptive, sustained or dysregulated glial activation promotes oxidative/nitrosative stress and neurotoxic inflammatory states; for example, chronic microglia-derived mediators can drive reactive astrocyte phenotypes that compromise neuronal support [5].

LPS challenge offers a practical, stimulus-defined approach to activate TLR4-coupled neuroinflammatory programmes, yet cross-study comparisons remain challenging. A major limitation is methodological variability in LPS itself (e.g., source/serotype, purification grade, chemotype, and potential contaminants), alongside heterogeneity in dose, exposure duration, and experimental context (cell density, serum conditions, co-treatments, route of administration in vivo) [6–8]. In addition, many studies sample readouts at timepoints that are poorly aligned with inflammatory kinetics, where early signalling events (minutes to hours) may be overinterpreted alongside later phenotype-level inflammatory outputs (hours–days) [9, 10]. Such inconsistency in reporting and sampling design can undermine reproducibility and weaken mechanistic attribution, thereby limiting the translational credibility of phytochemical screening claims.

This review focuses on isolated phytochemicals and chemically characterised natural products as candidate modulators of neuroinflammation evaluated in LPS-induced neuroinflammation models. This scope prioritizes candidates with a defined chemical identity and dose (supporting clearer PK/PD reasoning and mechanistic mapping), and it reduces ambiguity introduced by complex multi-component formulations in which composition, batch-to-batch consistency, and active constituents are not standardised or consistently reported. This review synthesises LPS-induced neuroinflammation models in phytochemical screening, with emphasis on study design, endpoint timing, and reporting practices that determine cross-study comparability and mechanistic interpretation. By framing LPS-responsive glial activation as an immunopharmacological stress-test rather than a disease-equivalent model, this review aims to support more rigorous interpretation of phytochemical effects on innate immune signalling, cytokine biology and inflammatory effector pathways.

Unlike previous reviews that mainly summarize LPS-induced neuroinflammatory pathways or phytochemical effects, this review focuses on how inflammatory kinetics should guide model selection, endpoint timing and interpretation of pharmacological responses. By distinguishing early signalling events from later mediator and phenotype-level outputs, the review proposes a practical framework for interpreting LPS-based phytochemical screening studies with greater mechanistic and translational precision.

## Literature search and framework development

This article is designed as a narrative review informed by a structured literature search. A structured literature search was conducted in PubMed, Scopus and Web of Science, supplemented by targeted retrieval in Google Scholar, to identify studies using LPS-induced neuroinflammation models for phytochemical screening. Searches combined controlled vocabulary and free-text terms with Boolean operators, using the following structure: (“lipopolysaccharide” OR “LPS” OR “endotoxin”) AND (“neuroinflammation” OR “microglia” OR “astrocyte” OR “gliosis” OR “Iba1” OR “GFAP” OR “cytokine”) AND (“phytochemical” OR “natural product” OR “polyphenol” OR “flavonoid” OR “terpenoid” OR “alkaloid” OR “phenolic acid”), with additional model terms applied where helpful (for example “BV-2”, “primary microglia”, “mouse”, “rat”, “zebrafish”/“Danio rerio”, “In vitro”, “In vivo”). The search was restricted to English-language articles published between 2010 and 2026. Titles/abstracts were screened followed by full-text assessment, and reference lists of eligible papers were manually searched to capture additional studies.

Studies were included if they used LPS to induce neuroinflammatory responses and tested an isolated phytochemical or well-characterized natural product with relevant inflammatory, oxidative-stress, glial, behavioural or mechanistic endpoints. For Tables 1 and 2, studies were selected from the eligible literature when sufficient methodological detail was reported to compare the model system, LPS exposure, sampling strategy and neuroinflammatory readouts. Tables 1 and 2 collectively represent 50 unique publications; Table 1 includes 36 unique publications and Table 2 includes 20, with six publications represented in both tables. Studies were excluded if LPS was not the main trigger, if no standalone phytochemical arm was included, or if neuroinflammation-relevant endpoints were lacking. Information from eligible studies providing sufficient methodological detail was mapped according to model type, LPS characteristics, exposure and sampling kinetics, control structure, endpoints, mechanistic interrogation and cross-model validation. Table 3 does not represent a separate study set but qualitatively synthesises recurring design and validation patterns identified across this evidence base into domains for model selection, evidence confidence and candidate progression. These study-derived features informed the framework, while the confidence levels, progression criteria and claim ceilings—including Level 3 as a point for considering in vivo progression—represent pragmatic, author-derived guidance rather than validated standards.

## In vitro LPS-induced neuroinflammation models

### Cellular platforms used to model LPS-induced neuroinflammation

LPS-driven phytochemical screening has been implemented most extensively in murine microglial systems, with murine microglial BV-2 cells emerging as a widely used platform for assessing soluble mediator release, transcriptional programmes and phosphorylation-resolved signalling readouts [11–16]. To enhance physiological relevance, primary microglia have been employed to capture broader activation phenotypes beyond mediator quantification, including proliferative responses, phagocytic activity and antigen-associated marker expression under LPS challenge [12, 17, 18]. Complementary murine microglial lines further expand the screening context, including N9 microglia, which are frequently used in nitrite/NO-centred paradigms and Iba1-linked activation readouts [19–22].

Beyond rodent microglia, human microglial and macrophage-like platforms have been incorporated to strengthen translational relevance. These include HMC3 and CHME3 microglia as well as THP-1-derived macrophages or reporter monocytes configured around NF-κB/AP-1-responsive outputs [17, 23–26]. Astroglial and mixed glial cell preparations have also been used to model LPS-responsive inflammatory programmes with glia-enriched activation markers [27–30]. Finally, neuronal systems, including HT22 neurons, SH-SY5Y neuroblastoma cells and dPC12 cells, further extend screening into downstream neurotoxicity-relevant phenotypes, capturing effects on viability, apoptosis-linked indices, and oxidative-stress signatures in the context of LPS exposure [31–34]. These platforms should not be considered interchangeable: immortalised microglial lines are useful for rapid screening, primary microglia provide stronger physiological relevance, and human or mixed-cell systems improve translational interpretation. Therefore, model choice should be aligned with the study aim, from high-throughput mediator screening to pathway validation and multicellular neurotoxicity assessment. Accordingly, findings from immortalised microglial lines should be interpreted mainly as screening-level evidence, whereas stronger mechanistic or translational claims require validation in primary, human-relevant, multicellular or in vivo models, as summarised in Tables 1 and 2. A summary of commonly used cell models, including their strengths, limitations and typical applications, is provided in Supplementary Table S1.

### LPS challenge design: serotypes, suppliers, dose and exposure kinetics

Across studies, reporting of LPS serotype and supplier is inconsistent; however, when specified, preparations are most commonly sourced from commercial vendors such as Sigma-Aldrich and related distributors [12, 15, 25]. Reported bacterial sources included *E. coli* serotypes O111:B4 and O55:B5, as well as *Salmonella typhimurium*, providing relatively well-defined inflammatory stimuli across multiple microglial and monocytic platforms [15, 25, 35–37].

**Fig. 1:**
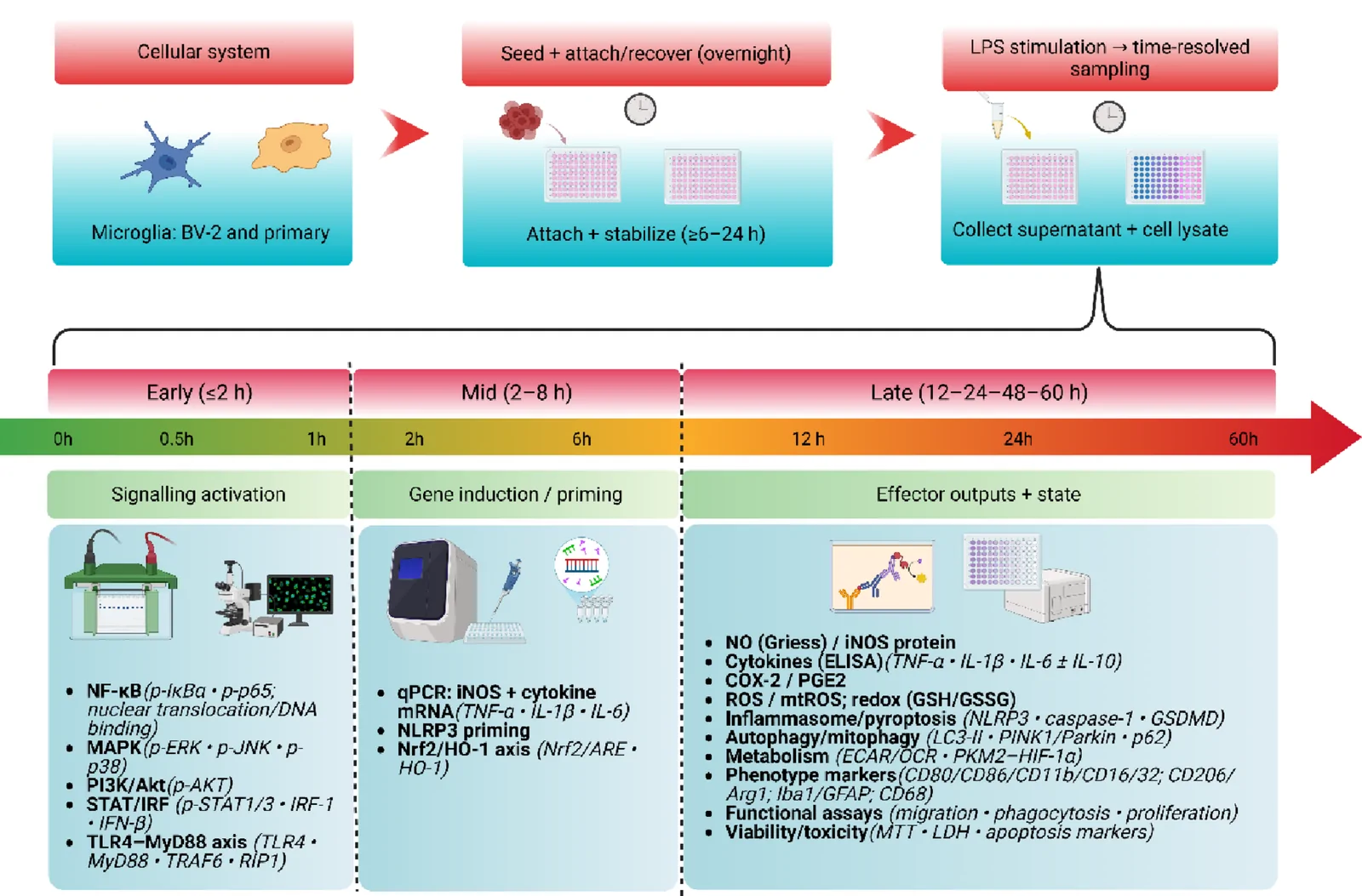
Establishing in vitro LPS-driven neuroinflammation models with time-aligned sampling and readouts. In vitro LPS neuroinflammation is typically established by selecting the cellular system, seeding and allowing attachment/stabilization (overnight), followed by LPS stimulation with time-resolved sampling. Cultures are commonly fractionated into supernatant (secreted mediators) and cell lysate (signalling, protein and RNA), with ± IFN-γ licensing used in some designs to increase inflammatory stringency. Early sampling (≤ 2 h) captures proximal signalling activation (NF-κB, MAPK, PI3K/Akt and STAT/IRF modules when measured), mid sampling (2–8 h) captures gene induction/priming (iNOS and cytokine mRNA; optional NLRP3 priming and Nrf2/HO-1 responses), and late sampling (12–24–48–60 h) captures effector outputs and cell state (NO/iNOS, cytokines, COX-2/PGE2, ROS/redox, and optional inflammasome, autophagy/mitophagy, metabolic and phenotype/functional readouts). Created in BioRender. Zhou, P. (2026) https://BioRender.com/n1qegmg

Dose selection spans several orders of magnitude and is typically tailored to the cellular platform and the primary readout, with commonly used microglial challenge conditions centred around 100 ng/mL and 1 µg/mL, alongside intermediate doses (200–500 ng/mL) and explicit multi-dose titrations [12, 38–41]. Exposure durations are often standardised at 24 h, yet many studies incorporate broader kinetic windows ranging from minutes to multiple days, and these designs enable capture of early pathway activation readouts (≤ 2 h), intermediate transcriptional and protein responses (2–8 h), and later inflammatory or phenotype-level outcomes (12–48 h or longer) [10, 26, 31, 37, 42, 43] (Fig. 1). Co-stimulation paradigms are occasionally used to intensify or refine the inflammatory state, including LPS combined with IFN-γ in microglia and LPS±Aβ designs in human microglial and neuronal models [10, 33, 42, 44]. LPS serotype, purity, dose and exposure duration can substantially influence inflammatory magnitude, signalling kinetics and endpoint interpretation. Early time points are more suitable for NF-κB/MAPK activation, whereas later time points better reflect cytokine release, iNOS/NO, ROS and phenotype-level inflammatory outputs.

### Readout landscape for LPS-induced neuroinflammatory activation

Nitric oxide (NO) biology dominates the readout landscape, with nitrite/NO quantification and inducible nitric oxide synthase (iNOS) induction serving as primary screening anchors across microglial and mixed-glial settings [9, 11, 29, 45–47]. Cytokine profiling, most commonly TNF-α, IL-1β and IL-6, is frequently assessed at mRNA and/or protein levels to define the inflammatory state against which phytochemical effects are benchmarked [12, 27, 48–51]. Prostaglandin signalling is also routinely incorporated, with PGE2 and COX-2 often measured as paired inflammatory outputs, typically alongside NO/iNOS and cytokine endpoints [9, 15, 36, 52, 53].

Oxidative-stress endpoints are commonly integrated with inflammatory readouts, including ROS/mtROS metrics, antioxidant status (for example GSH/GSSG) and broader redox-linked marker panels across both microglial and neuronal platforms [12, 15, 28, 34, 54, 55]. Cytotoxicity and viability assays are routinely included to contextualise anti-inflammatory effects and exclude non-specific suppression, including MTT, CCK-8, PI-based readouts, Calcein-AM/PI and LDH, with additional injury-associated outputs such as dsDNA release reported in macrophage contexts [26, 31–34, 36, 38]. In some study designs, functional coupling is assessed more directly by transferring microglial-conditioned media to neuronal cultures to quantify downstream effects on neuronal viability [53, 54].

A subset of studies extends beyond molecular outputs to phenotype and function, tracking microglial activation markers (for example Iba-1, CD11b, CD16/32, CD68 and CD80/CD86), migration, phagocytosis and morphology as screening-relevant endpoints [12, 24, 56–59]. Polarisation-associated markers (including Arg1, CD206, IL-10, IL-4 and TREM2) and metabolic readouts (ECAR/OCR shifts and glycolysis-associated metabolite signatures) are also deployed to position phytochemical effects within broader state transitions under LPS challenge [24, 41, 47, 56, 60]. Finally, cell-death-associated outputs, including apoptosis-associated markers and caspase readouts, further expand the response space, particularly in neuron-focused paradigms or microglial designs with more prominent injury components [31–34, 61].

### Mechanistic endpoints in LPS-induced models

**Fig. 2:**
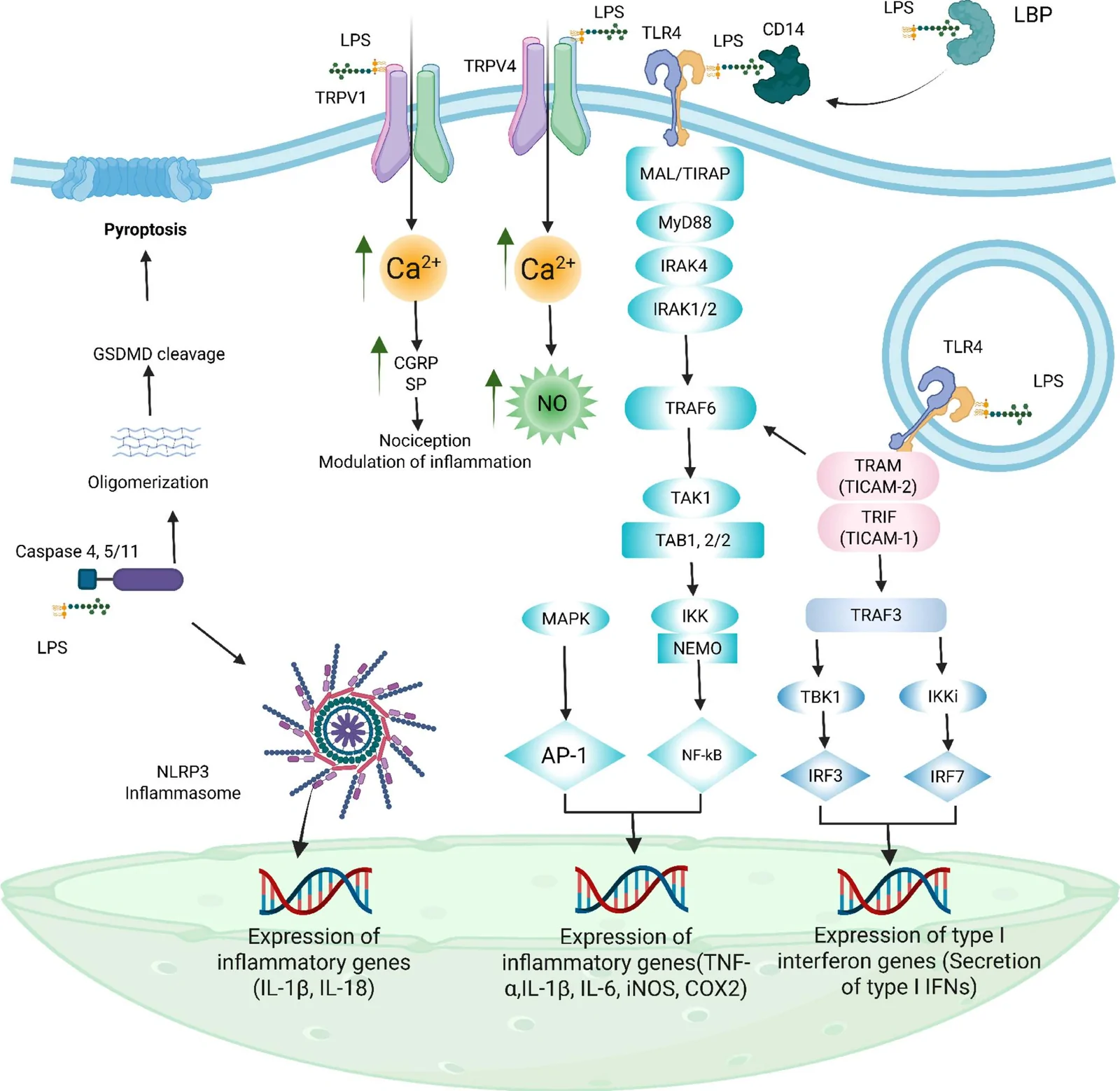
Molecular mechanisms and kinetics of cellular responses to LPS. LPS is captured by LPS-binding protein (LBP) and transferred to CD14, which presents LPS to TLR4 to initiate receptor signalling at the plasma membrane. MyD88-dependent signalling proceeds *via* MAL/TIRAP → MyD88 → IRAK4/IRAK1/2 → TRAF6 → TAK1 associated with Tables 1 and 2/Table 3, activating the IKK complex (IKK/NEMO) and MAPK pathways. These branches drive nuclear transcriptional programmes through NF-κB and AP-1, promoting expression of inflammatory mediators (for example TNF-α, IL-1β, IL-6, iNOS, COX-2). In parallel, TRIF-dependent (endosomal) signalling is mediated by TRAM (TICAM-2) → TRIF (TICAM-1) → TRAF3, activating TBK1 and IKKε (IKKi) to induce IRF3/IRF7-dependent type I interferon gene expression and interferon secretion. Non-canonical inflammasome signalling is triggered by cytosolic LPS sensing through caspase-4/5 (human) or caspase-11 (mouse), leading to gasdermin D (GSDMD) cleavage, pore formation/oligomerization, and pyroptosis, with crosstalk to NLRP3 inflammasome activation and maturation of IL-1β and IL-18. Ion-channel modules (TRPV1/TRPV4) contribute to Ca²⁺ influx, linking LPS exposure to NO production, nociceptive neuropeptides (for example CGRP and substance P), and modulation of inflammatory tone. Created in BioRender. Zhou, P. (2026) https://BioRender.com/0wv699l

Mechanistic interrogation in LPS-induced neuroinflammation studies most often converges on a relatively restricted set of signalling modules (Fig. 2), which are repeatedly used to position compound effects beyond simple changes in mediator release [62]. NF-κB-centred signalling represents the most frequently examined mechanistic layer in LPS-induced models, assessed through p65 phosphorylation, nuclear translocation, DNA-binding activity, pathway reporter outputs and upstream IκB/IKK dynamics across microglial, astroglial and monocytic platforms [10, 12, 18, 23, 25, 37, 50, 63]. In some studies, receptor–adaptor context is made explicit, through assessment of TLR4 and MyD88 in microglial and neuronal settings, alongside additional signalling nodes, including TRAF6/RIP1 and Cezanne, positioned as modulators of NF-κB activity [31, 38, 47, 49, 51]. Selected studies further extend innate immune mapping beyond canonical NF-κB to include TBK1–IRF3 signalling and, in some contexts, STING-associated responses alongside NF-κB phosphorylation readouts, thereby enabling broader placement of phytochemical effects across inflammatory signalling nodes [61].

Mechanistically, LPS-induced neuroinflammation can be organized into core inflammatory signalling and secondary regulatory pathways. The core axis includes LPS–TLR4/CD14/MD-2 recognition, MyD88/TRIF adaptor signalling, NF-κB activation, MAPK phosphorylation and downstream induction of TNF-α, IL-1β, IL-6, iNOS/NO and COX-2/PGE2. Secondary regulatory modules, including Nrf2/HO-1, PI3K/Akt, AMPK, JAK/STAT, NLRP3 inflammasome activation, mitophagy and metabolic rewiring, modulate the magnitude, duration or resolution of this core inflammatory response rather than acting as primary LPS-sensing pathways.

In parallel, MAPK cascades (ERK, JNK and p38, together with upstream regulatory nodes) and PI3K/Akt-linked signalling are commonly profiled as complementary mechanistic readouts, with some studies incorporating AMPK modules and broader kinase phosphorylation panels [13, 15, 27, 36, 39, 52, 55, 64].

Inflammasome-associated mechanisms are resolved in a subset of paradigms through NLRP3- and caspase-1-linked outputs, including GSDMD-N formation and IL-1β processing [27, 40, 65]. By contrast, autophagy-mitophagy and oxidative-stress response pathways are better interpreted as secondary mechanistic layers that modulate LPS-induced response kinetics, rather than as primary LPS-sensing modules. Accordingly, markers such as LC3-II, PINK1, Parkin and p62, together with Nrf2/ARE-associated effectors including HO-1 and NQO1, are increasingly incorporated to refine interpretation of phytochemical actions in relation to mitochondrial integrity, stress adaptation, signal persistence and inflammatory resolution [64]. This expanded layer is further supported by broader antioxidant enzyme panels, complementary assessment of NOX2/NOX4 in primary microglia, and measurement of mitochondrial injury or mitophagy-related features in neuronal models [15, 24, 34, 42, 66].

Finally, several studies frame LPS-driven states through metabolism- and fate-linked nodes, including PKM2 phosphorylation and nuclear translocation linked to HIF-1α, ECAR/OCR shifts, Smad3/cyclin D3 phosphorylation and apoptosis marker panels, thereby connecting inflammatory activation to downstream functional impairment [31, 34, 41, 61, 67]. Experimental design features and mechanistic readouts are summarized in Table 1.

**Table 1:** In vitro cellular platforms, LPS challenge design and mechanistic readouts used for phytochemical screening in LPS-induced neuroinflammation models

Cell line/primary culture (species)	LPS serotype & supplier	LPS dose (ng/mL or µg/mL)	Exposure duration (h)	Readouts (inflammatory + mechanistic)	References
BV2 microglia (mouse)	NR, PeproTech	100 ng/mL	24	↑TNF-α, ↑IL-1β, ↑IL-6, ↑NO; ↑TNF-α/IL-12p70 mRNA; ↑iNOS, ↑CD16/CD11b/CD86; ↑TGF-β; TLR4/NF-κB p65 and STAT1 activation; microglial M1→M2 shift with baicalein	[48]
BV2 microglia (mouse)	NR, Sigma-Aldrich	1 µg/mL	0.25, 1, 2, 6, 24	↑iNOS, ↑NO; ↑HO-1, ↑Nrf2/ARE	[42]
BV2 microglia (mouse)	NR, Sigma-Aldrich	100 ng/mL	1, 6, 12, 20	LPS: ↑NO; ↑TNF-α, ↑IL-1β, ↑IL-6; ↑iNOS (protein/mRNA); ↑NF-κB p65 binding	[50]
BV2 microglia (mouse)	NR, Sigma	1 µg/mL	24	↑NO; ↑IL-1β, ↑IL-6, ↑iNOS, ↑CD16/32; ↓IL-4, ↓IL-10, ↓Arg-1, ↓CD206; TREM2↓, TLR4↑, NF-κB p65/IκBα activation	[47]
BV2 microglia (mouse)	NR, Sigma-Aldrich	100 ng/mL (with IFN-γ 10 U/mL)	0.17, 1, 3, 6, 16, 24	↑NO; ↑iNOS; ↑TNF-α, ↑IL-1β, ↑IL-6; ↑MIP-1α, ↑MCP-1, ↑IP-10; ↑NF-κB p65 nuclear translocation; ↑IFN-β, ↑phospho-STAT-1, ↑IRF-1; ↑JNK/ERK/p38 phosphorylation	[10]
BV2 microglia (mouse)	*E. coli* 055:B5, Sigma	100 ng/mL	6, 16, 18, 24	LPS: ↑NO, ↑PGE2; ↑TNF-α, ↑IL-6, ↑IL-1β; ↑iNOS, ↑COX-2; ↓GSH, ↑GSSG/GSH; ↑NF-κB p65 nuclear translocation, ↑IκBα phosphorylation	[15]
BV2 microglia (mouse)	NR, NR	500 ng/mL	1, 18	↑NO, ↑PGE2, ↑TNF-α, ↑IL-1β; ↑iNOS, ↑COX-2; ↑IκB-α phosphorylation, ↑NF-κB p65/p50 nuclear translocation and DNA-binding; microglia-mediated	[53]
BV2 microglia (mouse)	*Salmonella typhimurium*, Sigma Aldrich	100 ng/mL	1, 4, 6, 24	↑TNF-α, ↑IL-6, ↑IL-1β, ↑NO, ↑PGE2; ↑iNOS, ↑COX-2; ↑NF-κB DNA binding and luciferase activity; ↑p-IκBα, ↑p-p65, ↑nuclear p65	[37]
BV2 microglia (mouse)	NR, NR	100 ng/mL	24	↑CD11b (microglial activation); ↑TNF-α, ↑IL-6 (secretion, mRNA); ↑NO; ↑iNOS (mRNA, protein); ↑p-IκBα, ↓IκBα; ↑nuclear NF-κB p65, ↓cytoplasmic p65; ↑ROS; ↑NADPH oxidase activity, ↑gp91phox)	[54]
BV2 microglia (mouse)	*E. coli* O111:B4, Sigma	1 µg/mL	24	↑NO; ↑IL-1β, ↑IL-6, ↑TNF-α; ↑TLR4, ↑MyD88, ↑p-p65/p65	[51]
BV2 microglia (mouse)	*E. coli* O111:B4, Sigma-Aldrich	25, 50, 100, 250, 500, 1000 ng/mL	24	↑NO; ↑IL-1β, ↑TNF-α; ↓IL-10, ↓IL-4; ↑NOS2, ↓Arg1; ↑glycolysis (↑ECAR, ↑G6P, ↑3PG, ↑PEP, ↑Pyr, ↑Lac; ↓OCR); ↑PKM2 phosphorylation/nuclear translocation; ↑HIF-1α	[41]
BV2 microglia (mouse)	NR, MedChemExpress	1 µg/mL	6	↑TNF-α, ↑IL-1β, ↑IL-6; ↓IL-10, ↓TGF-β; ↑CD68, ↑iNOS; ↓CD206, ↓Arg1; ↑STING, ↑p-TBK1, ↑p-IRF3, ↑p-NF-κB; ↓Bcl-2; ↑Bax, ↑cleaved caspase-3, ↑caspase-9; ↑apoptosis (TUNEL)	[61]
BV-2 microglia (mouse)	NR, Sigma-Aldrich	100 ng/mL	24, 48	↑IL-1β, ↑IL-6, ↑TNF-α; ↑iNOS/NO; ↑ROS/mtROS; NF-κB (IKKβ/p65) activation; ↑NLRP3 inflammasome (NLRP3, cleaved caspase-1, GSDMD-N, IL-1β); ↓mitophagy (LC3-II, PINK1/Parkin, p62)	[12]
BV-2 microglia (mouse)	NR, NR	2 µg/mL	24	LPS: ↑NO; ↑iNOS (protein/mRNA); ↑TNF-α, ↑IL-1β (mRNA); ↑p-NF-κB p65; ↑p-AKT	[13]
BV-2 microglia (mouse)	*E. coli* Serotype O55:B5, Sigma-Aldrich	1.0 µg/mL	4, 24	↓cell viability (MTT); ↑NO, ↑PGE2; ↑iNOS, ↑COX-2; ↑TNF-α, IL-1β, IL-2, IL-6, IL-12, IFN-γ; ↑CCL21, CCL5/RANTES, galectin-3; activation of NF-κB p65/IκBα, PI3K/Akt, p38/ERK/JNK MAPK	[36]
BV-2 microglia (mouse)	NR, Sigma-Aldrich	1 µg/mL	0.5, 12, 24	↑NO; ↑TNF-α/IL-1β/IL-6/IL-10; ↑iNOS, ↑COX-2; ↑p-ERK1/2, ↑p-JNK, ↑p-p38; ↑NF-κB p65 nuclear translocation	[17]
BV-2 microglia (mouse)	NR, NR	4 µg/mL	3	↑IL-1β, ↑TNF-α mRNA; ↑p-IKBα, ↑p-NF-κB p65	[43]
BV-2 microglia (mouse)	NR, NR	100 ng/mL	0.5, 1, 6, 24	↑NO; ↑TNF-α, ↑IL-1β; ↑iNOS, ↑COX-2; ↑NF-κB activity; ↑p-JNK (MAPK)	[68]
BV-2 microglia (mouse)	NR, NR	1 µg/mL	22	↑NO, ↑PGE2, ↑TNF-α, ↑IL-6; ↑p-Smad3, ↑p-cyclin D3	[67]
BV-2 microglia (mouse)	NR, NR	100 ng/mL	24	↑NO; ↑Iba-1; ↑iNOS, ↑COX-2	[59]
BV-2 microglia (mouse)	NR, Sigma-Aldrich	200 ng/mL	24, 18, 6, 0.5	↑NO; ↓cell viability (MTT); ↑iNOS, ↑COX-2; ↑IL-6, ↑TNF-α; ↑p-p38, ↑p-ERK, ↑p-JNK; ↑p-IκB-α, ↑NF-κB p65 nuclear translocation	[39]
BV-2 microglia (mouse)	*E. coli*, Sigma-Aldrich	500 ng/mL (with 0.5 ng/mL IFNγ)	24	LPS/IFNγ: ↑IL-2, ↑IL-4, ↑IL-6, ↑IL-10, ↑IL-17a (protein); ↑IL6, ↑CFB, ↑C3, ↑CXCL3, ↑CCL5 (NFκB targets)	[44]
BV-2 microglia (mouse)	NR, Sigma-Aldrich	100 ng/mL	24, 16, 6, 3, 1, 0.17	↑NO; ↑iNOS; ↑TNF-α, ↑IL-1β, ↑IL-6; ↑MIP-1α, ↑MCP-1, ↑IP-10; ↑NF-κB p65 nuclear translocation; ↑IFN-β, ↑phospho-STAT-1, ↑IRF-1; ↑phospho-JNK, ↑phospho-ERK, ↑phospho-p38	[10]
C6 astroglioma (rat)	NR, Sigma-Aldrich	1 µg/mL	24	↑TNF-α, ↑IL-6, ↑NO; ↑ROS; ↑NOS2 (iNOS)	[28]
C8-D1A astrocytes (mouse)	NR, Sigma-Aldrich (USA)	1 µg/mL	18, 24	↑TNF-α, ↑IL-1β, ↑IL-6, ↑NO; ↑iNOS, ↑MMP-9; ↑NLRP3, ↑caspase-1; ↑ROS, ↑[Ca²⁺]i; activation of NF-κB p65, STAT3 and MAPKs (AKT, MEK/ERK, p38, JNK)	[27]
CHME3 microglia (human)	NR, NR	1 µg/mL	0.5	↓NF-κB pathway inhibitor (biosensor); ↑NF-κB activity	[23]
dPC12 pheochromocytoma cells (rat)	NR, NR	50 µg/mL	4	↑ROS (DCFH-DA, CellROX, MitoSOX); ↓mitochondrial membrane potential (JC-1); mitochondrial fragmentation; ↑mitophagy/autophagy; ↑TLR-4, ↑MyD88, ↑iNOS; ↑TNF-α, ↑IL-1β, ↑IL-6; ↓IL-4, ↓IL-10; ↑NF-κB p65 nuclear translocation; ↓Nrf2, ↓HO-1, ↓NQO1, ↓SOD, ↓CAT; ↑Bax, ↑Bad, ↑caspase-3; ↓Bcl-2	[34]
HMC3 microglia (human)	NR, Sigma-Aldrich	NR	12, 24	↑IL-6, ↑COX-2, ↑IL-10; ↑p-ERK1/2, ↑p-JNK, ↑p-p38; ↑NF-κB p65 nuclear translocation	[17]
HMC3 microglia (human)	NR, Sigma-Aldrich	1 µg/mL	24	↑TNF-α, ↑IL-6, ↑IL-1β; ↑CD80, ↑CD86, ↑CD68, ↑iNOS; ↑TLR4, ↑MyD88; ↑ROS	[24]
HMC3 microglia (human)	NR, Sigma	0.1–100 µg/mL (toxicity screen; selected toxic dose used for assays)	12, 24, 48	↑NO, ↑LDH; ↑TNF-α, ↑IL-1β, ↑IL-6; ↑TLR4, ↑AKT1, ↑MAPK1/3 (ERK1/2), ↑NF-κB, ↑PTGS2; ↑iNOS	[33]
HT22 hippocampal neurons (mouse)	*E. coli* O111:B4 (Materials); 055:B5 (cell culture), Sigma-Aldrich	25, 50, 100, 250, 500, 1000, 1500 µg/mL	12, 24, 36, 48, 60	↑TNF-α, ↑IL-6, ↑IL-1β; ↑TLR4; ↑p-NF-κB, ↑p-IκBα, ↓IκBα; ↑apoptosis, ↑Bax, ↓Bcl-2	[31]
HT22 hippocampal neurons (mouse)	NR, NR	1 µg/mL	24	↑apoptosis markers; ↑inflammatory cytokine mRNA	[32]
N9 microglia (mouse)	NR, Sigma-Aldrich	300 ng/mL	24	↑Iba1; ↑NO; ↑TNF-α	[20]
Primary cortical glial cells (rat)	*E. coli*, Sigma Aldrich	1 µg/mL	1, 24	↑NO; ↑NF-κB phosphorylation; ↑GFAP, ↑Iba1; astrocyte and microglia activation	[29]
Primary microglia (mouse)	NR, Sigma-Aldrich	100 ng/mL	24	↑IL-1β, ↑IL-6, ↑TNF-α; ↑proliferation; ↑phagocytosis; ↑CD68	[12]
Primary microglia (mouse)	NR, Sigma-Aldrich	200 ng/mL	12	LPS/IFN-γ: ↑NO	[42]
Primary microglia (mouse)	*E. coli* O55:B5, Sigma	1 µg/mL	2, 24	↑TNF-α, ↑IL-1β; ↑iNOS, ↑COX-2; ↑NO, ↑PGE2; ↑NOX2, ↑NOX4; ↑ROS	[66]
Primary microglia (mouse)	NR, Sigma	1 µg/mL	0.5, 1, 24	↑TNF-α, ↑IL-1β, ↑IL-6; ↑NO, ↑PGE2; ↑iNOS, ↑COX-2; ↑IκBα phosphorylation, ↑NF-κB p65 nuclear translocation; ↑p-JNK, ↑p-p38; ↑p-JAK1, ↑p-STAT1, ↑p-STAT3	[18]
Primary microglia (rat)	*Salmonella typhimurium*, Sigma Aldrich	10 ng/mL	4, 24	↑TNF-α, ↑IL-6, ↑IL-1β, ↑PGE2; ↑COX-2	[37]
SH-SY5Y neuroblastoma (human)	NR, Sigma	0.1–100 µg/mL (toxicity screen; selected toxic dose used for assays)	12, 24, 48	↑NO, ↑LDH; ↑APP, ↑ASC, ↑p-Tau, ↑caspase-3, ↑STAT3; ↑α-syn	[33]
THP-1 macrophages (human)	*E. coli* O111:B4, Sigma-Aldrich	1 µg/mL	24	↑TNF-α, ↑IL-1β	[25]
THP-1-derived macrophages (human)	NR, Cell Signaling Technology	100 ng/mL	3	↑NO; ↑dsDNA release; ROS unchanged	[26]
THP-1-XBlue™-MD2-CD14 monocytes (human)	*E. coli* O111:B4, Sigma-Aldrich	1 µg/mL	0.5, 24	↑NF-κB/AP-1 (SEAP); ↑IκBα degradation; ↑p-ERK1/2, ↑p-p38, ↑p-JNK	[25]

Abbreviations: NR, not reported

## In vivo LPS-induced neuroinflammation models

### Animal cohorts used to model LPS-induced neuroinflammation

Across in vivo LPS paradigms, cohort characteristics (species, strain, age and sex) represent an important contextual layer for interpreting neuroinflammatory readouts and pharmacological effects. LPS-driven neuroinflammation was modelled predominantly in mice and rats, with additional applications in adult zebrafish, zebrafish larvae and embryos [12, 69–73]. Rodent studies most used C57BL/6 or C57BL/6J mice, alongside ICR, BALB/c and Swiss mice, and Wistar or Sprague-Dawley rats [12, 69, 74–79].

Age reporting was centred mainly on young adult animals (for example, 5–8 weeks through to ~ 3 months, and 60 days in some mouse studies, with 10-18-week windows in several rat cohorts), while a subset used early-life exposure paradigms including neonatal rats (postnatal day 14) and mice dosed during postnatal day 3 to postnatal day 5 [12, 32, 49, 56, 66, 78, 80, 81]. Sex reporting also represents an important limitation. Most rodent studies used male animals, with fewer mixed-sex designs and limited explicit sex-stratified analysis. Because immune signalling, microglial activation and inflammatory mediator production can be sex-dependent, underrepresentation of female animals may limit generalisability and contribute to inter-study variability in LPS-induced neuroinflammatory outcomes [32, 38, 69, 71, 72, 76].

### LPS challenge design In vivo: route, dose, schedule, and serotype reporting

Systemic intraperitoneal administration predominated across in vivo models, encompassing both single-dose challenges (for example 100 µg/kg, 600 µg/kg, 0.83–0.85 mg/kg, 1 mg/kg, 2 mg/kg, 5 mg/kg and 6 mg/kg) and repeated daily schedules (for example 0.25 mg/kg for 1 week, 0.5 mg/kg for 6 days, 0.75 mg/kg for 7 days, 1 mg/kg for 5 days, 167 µg/kg for 7 days, 250 µg/kg for 7 days, 2 mg/kg for 3 days, and 250 µg/kg for 14 days) [31, 38, 41, 56, 69, 70, 76, 77, 81–83]. Systemic LPS exposure should not be interpreted as isolated CNS neuroinflammation because it also engages peripheral immune and physiological responses; stronger CNS attribution therefore requires brain-region inflammatory, glial or neuronal readouts alongside systemic or functional outcomes. Central delivery routes were also used, including intracerebroventricular LPS administration at 40 µg and 150 µg/5 µL, and an intracerebroventricular 10 µg/5 µL paradigm, as well as zebrafish larval intracerebroventricular injection (1 nL of 5 mg/mL) and larval intracerebral dosing at 2.5 mg/mL (injection volume not reported) [72, 74, 79, 84]. Less common exposure routes included subcutaneous exposure in neonatal mice (50 µg/kg once daily during PND3-5), an intratracheal dosing model (0.003 g/kg single dose), and a waterborne embryo exposure (100 ng/mL for 24 h) [73].

When reported, *E. coli* serotype information included O55:B5, O111:B4 and O26:B6, with suppliers such as Sigma and Beyotime noted in several entries; however, many studies still did not report serotype even when an *E. coli* origin and/or supplier was specified [49, 70, 73, 75]. Reporting of route and injection frequency was also incomplete in some paradigms, including a single-dose 0.83 mg/kg study in which route was not reported and a 0.4 mg/kg exposure described as occurring “for last week” without route or injection-frequency details [41, 75].

### Post-challenge timeline and sampling strategy

Sampling windows ranged from acute post-challenge assessments to long-latency follow-up. Acute designs commonly included 4–24 h behavioural testing with tissue collection at ~ 24 h, as well as 24 h and 48 h endpoints, and combined 6–24 h behavioural and sampling sequences. These are complemented by multi-day and delayed schedules, including 2 days, 7 days and 30 days’ assessments, as well as extended follow-up at 90–97 days following neonatal exposure [41, 71, 74, 77, 78].

In repeated-dose paradigms, sampling was frequently anchored to completion of the dosing regimen, commonly described as “1 day after last LPS” or as “day 7” after behavioural testing, although in several studies the precise interval from the final LPS exposure was not reported [31, 38, 66, 70, 82, 85, 86].

Behavioural testing and tissue collection were coordinated across acute, cognitive and longer-term neurodevelopmental schedules [28, 32, 56, 79, 80, 87]. Rodent sampling focused mainly on the hippocampus and cortex, whereas zebrafish studies examined brain- or head-level cytokine and neuronal outcomes [12, 41, 69, 70, 72, 74, 81, 84]. The design features of in vivo LPS neuroinflammation models, including rodent regional sampling and challenge architecture across species and routes, are summarised in Supplementary Figure S1.

### Readout landscape in vivo: neuroinflammatory activation and mechanistic positioning

Across in vivo LPS paradigms, readout architecture is designed to link neuroimmune activation with tissue-level injury and pathway engagement, enabling mechanistic positioning beyond cytokine changes alone. Readout selection consistently prioritised glial activation states and inflammatory mediators, with microglial activation commonly assessed using ionized calcium-binding adaptor molecule 1 (Iba-1)/ allograft inflammatory factor 1(AIF-1) markers, changes in microglial morphology or area, and cluster of differentiation (CD68) or CD86/CD163 co-labelling to position activation phenotypes within defined regions such as hippocampus (including DG/CA1/CA3) and cortex [12, 41, 66, 74, 76, 81, 84]. GFAP-based astroglial measures were often paired with neuronal integrity and apoptosis markers [28, 31, 74, 76, 78].

Oxidative and redox-associated measures were frequently integrated, including TBARS/MDA, reactive species, nitrite, total thiols, SOD/CAT and GSH/GST, and were sometimes paired with cholinesterase activity (AChE and/or BChE) and neurochemical readouts (for example GABA/Glu/5-HT/DA), broadening sensitivity to wider inflammatory physiology [41, 69–71, 82, 85].

Mechanistic positioning was most often anchored to defined signalling nodes and effector programmes explicitly assayed in vivo, including NF-κB activation (phosphorylation, nuclear translocation, DNA-binding and/or downstream targets), TLR4 readouts, inflammasome-related markers (NLRP3 and active caspase-1) and pathway-linked regulators such as Nrf2/HO-1/Keap1, MAPK phosphorylation (ERK/p38/JNK), PI3K/Akt signalling and mitophagy-associated PINK1 [12, 38, 74, 75, 81, 82, 86]. Experimental design features and readout parameters are summarized in Table 2.

**Table 2:** In vivo LPS-induced neuroinflammation models across species: dose/route, time course and neuroinflammatory changes

Species	Age	Sex	LPS administration	Sacrifice	Neuroinflammatory changes	References
AB (Danio rerio, zebrafish larvae)	5 dpf	U	*E. coli* (O55:B5, Sigma) i.c. 2.5 mg/mL (single dose); volume NR	24 h	↑NO and ROS (larval heads); microglial activation (↑ Iba1 protein); ↑ iNOS, TNF-α, IL-6, IL-1β mRNA.	[88]
BALB/c (mouse)	7–8 w	M	*E. coli* (O111:B4) i.p. 0.75 mg/kg (every day / 7 day)	NR	↑ hippocampal IL-6, IL-1β, TNF-α, NF-κB and GFAP mRNA; marked microglial and astroglial activation (↑ Iba1 + and GFAP+ cells in CA1 & CA3); neuronal loss in CA1; gamma-oryzanol treatment attenuated LPS-induced cytokine upregulation, glial activation and CA1 neuronal loss.	[76]
C57BL/6 (mouse)	NR	M	LPS i.c.v. 40 µg (single dose); serotype NR	24 h	LPS: ↑NO levels (brain); ↑ iNOS and IL-1β mRNA (cortex); Microglial activation: ↑ Iba-1 + cells (hippocampus DG and cortex); Neuronal damage: ↓ NeuN immunoreactivity (hippocampus DG and cortex); ↑ NLRP3 inflammasome formation (cortex).	[74]
C57BL/6 (mouse)	8–12 w	M	*E. coli* (O111:B4, Sigma) i.p. 0.25 mg/kg (once a day / 1 w)	NR (brain collected after completion of behavioural testing; exact interval from last LPS NR)	Microglial activation: ↑ Iba-1-positive cells (hippocampus); ↑ IL-1β and IL-6 levels; ↑ TLR4, p-IκBα and p-NF-κB expression (hippocampus); ↑ neuronal apoptosis: ↑ TUNEL-positive cells; ↑ Bax, ↓ Bcl-2; neuronal damage: ↓ Nissl bodies, sparse neuronal arrangement; impaired neurogenesis: ↓ EdU⁺/NeuN⁺ cells (hippocampus).	[31]
C57BL/6 (mouse)	7–8 w	M	LPS i.p. 0.5 mg/kg (every day / 6 days); serotype NR	1 day (after last MWM trial)	Microglial activation: ↑ Iba-1⁺ cells (hippocampus, cortex); neuronal loss: ↓ NeuN⁺ neurons; pro-inflammatory mediators: ↑ iNOS, IL-1β, TNF-α, IL-6; NF-κB activation: ↑ p-IκBα, nuclear p65, ↓ IκBα.	[84]
C57BL/6 (mouse)	6–8 w	M	*E. coli* (O111:B4) 0.83 mg/kg (single dose); route NR	4 h, 7 h and 24 h (behaviour tests); 24 h (tissue collection)	Microglial activation with M1 shift (↑ Iba-1, ↑ CD86+/Iba1+, ↓ CD163+/Iba1+); ↑ IL-1β, TNF-α and ↓ IL-10, IL-4 (serum, hippocampus, mPFC, amygdala); ↑ PKM2 in hippocampal microglia; neuronal/synaptic injury (↓ NeuN, ↓ SynI, ↓ dendritic spines, synaptic ultrastructural damage); neurotransmitter imbalance (↓ GABA, ↑ Glu, 5-HT, DA)	[41]
C57BL/6 (mouse)	8 w	M	*E. coli* (O111:B4, Beyotime) i.p. 6 mg/kg (single dose)	24 h after LPS	Microglial activation: ↑ Iba1 + microglia and ↑ Iba1 fluorescence intensity with activated morphology in hippocampal CA1.	[49]
C57BL/6 (mouse)	5–6 w	U	LPS i.p. 0.25 mg/kg (every day / 7 days); serotype NR	NR	Hippocampus and cortex: ↑ TNF-α and IL-1β (≈ 2-4-fold vs. control); neuroinflammation-associated neuronal damage (↓ Nissl-positive neurons, loss of Nissl staining, blurred cell boundaries); ar-turmerone and derivatives (7a, 7c, 7f, 9b) ↓ TNF-α/IL-1β toward control levels and restored Nissl-positive neurons/morphology	[89]
C57BL/6J (mouse)	3 m	M	LPS i.p. 1 mg/kg (every day / 5 days); serotype NR	2 days	Microglial activation: ↑ IBA-1 + microglia density and amoeboid morphology (hippocampus, cortex); ↑ CD68 fluorescence intensity in IBA-1 + cells (hippocampus). Inflammasome/mitophagy: ↑ NLRP3, ↑ active caspase-1, ↑ mature IL-1β protein (hippocampus); ↓ PINK1 (hippocampus).	[12]
C57BL/6J (mouse)	8 w	M	LPS i.p. 100 µg/kg (single dose); serotype NR	24 h (microglial morphology, TST); 30 h (FST); 48 h (SPT)	Microglial activation with process retraction/amoeboid morphology in prefrontal cortex; behavioural abnormalities: ↑ immobility time in TST and FST; ↓ sucrose preference (SPT).	[56]
C57BL/6J (mouse)	2 m	M	*E. coli* (O55:B5, Sigma) i.p. 2 mg/kg (every day / 3 days)	1 day	↑ TNF-α, IL-1β, PGE2 and nitric oxide (hippocampus); ↑ p-NF-κB p65; ↑ COX2, iNOS, NOX2, NOX4 (hippocampal tissue); Microglial activation: ↑ Iba1⁺ cell number and soma size in hippocampus.	[66]
C57BL/6J (mouse)	3–5 d	M; F	*E. coli* (O111:B4, Sigma) s.c. 50 µg/kg (once daily / PND3-5)	24 h and 5 w after EdU given after last LPS (EdU assays); 4 w after PIA for MWM/NORT; 6–8 w of age for hippocampal analyses	Hippocampal neuronal and synaptic injury: ↓ NeuN⁺ neurons; ↓ EdU⁺/EdU⁺NeuN⁺ cells; ↓ PSD95, synaptophysin and spine density; ↓ BDNF/CaMKII/Nr2b; ↑ Bax and active caspase-3; ↓ BCL2; ↑ EZH2 and H3K27me3 (Bcl2/BDNF promoters).	[32]
C57BL/6J (mouse)	8 w	M	LPS (Sigma) i.p. 250 µg/kg (every day / 14 days); serotype NR	after 14 days of LPS treatment (end of course; exact interval NR)	↑ IL-1β, IL-6, TNF-α, iNOS in cortex/hippocampus; ↓ IL-4, IL-10, TGF-β; microglial activation (↑ Iba-1 + area, hyper-ramified morphology); hippocampal (CA1/CA3/DG) and cortical neuronal injury with neuronal loss, disorganized cytoarchitecture and ↑ TUNEL+ cells; apoptosis shift (↑ Bax, ↑ caspase-3, ↓ Bcl-2); activation of TLR4/PI3K/AKT/NF-κB signalling.	[83]
Danio rerio (zebrafish embryos)	24–48 hpf	U	*E. coli* (O55:B5, Sigma) 100 ng/mL (waterborne / 24 h)	24 h LPS exposure	↑ NO production in LPS-treated embryos (↑ DAF-FM fluorescence).	[73]
Danio rerio (zebrafish)	3 m	M; F	LPS (Sigma) i.p. 1 mg/kg (single dose); serotype NR	7 days after LPS	↑ TNF-α and IL-1β in brain; ↑ lipid peroxidation (LPO), nitrite and AChE; ↓ GSH; histology: disarranged cell layers and pyramidal neuronal cell loss.	[71]
Rat	17–18 w	M	LPS i.p. 0.83–0.85 mg/kg (single dose); serotype NR	24 h	↑ IL-6, TNF-α, IL-17 (hippocampus) and ↑ TNF-α (prefrontal cortex); microglial activation (↑ AIF-1 + cells in PFC and CA1); ↑ NLRP3 inflammasome, NF-κB and iNOS immunoreactivity in hippocampus and PFC.	[81]
Swiss (mouse)	60 d	M	*E. coli* (O55:B5, Sigma) i.p. 250 µg/kg (every day for 7 days)	1 day after last LPS (after behavioural tests)	LPS: ↑ TNF-α levels in cerebral cortex, striatum and hippocampus; ↑ reactive species (RS) and nitrite; ↑ TBARS in striatum; ↓ total thiol content (cortex, hippocampus); ↓ SOD and CAT activities (cortex, striatum, hippocampus).	[70]
Tg(mpo: EGFP) (Danio rerio, zebrafish larvae)	5 dpf	U	LPS (Beyotime) i.c.v. 1 nL of 5 mg/mL into brain ventricles (single dose); serotype NR	24 h after LPS (24 hpi)	↑ neutrophil recruitment in brain; ↑ TNF-α and IL-1β mRNA in head; ↑ NO in brain; raphe neuron damage (↓ Ra neuron length and fluorescence); locomotor deficit (↓ total distance moved).	[72]
Wistar (rat)	10–12 w	M	LPS i.p. 1 mg/kg (single dose); serotype NR	48 h	↑ TNF-α, IL-1β, IL-6 protein levels (hippocampus); ↑ TNF-α, IL-1β, IL-6 mRNA expression (hippocampus); ↑ ROS and TBARS/MDA; ↓ SOD and CAT activities; ↓ GSH levels; ↓ SOD and CAT mRNA expression; ↑ AChE and BChE activities (hippocampus); cognitive impairment in MWM (↑ escape latency; ↓ entries/time in target quadrant).	[69]
Wistar (rat)	NR	M	LPS i.p. 5 mg/kg (single dose); serotype NR	24 h and 7 days	Astroglial/microglial activation: ↑ GFAP; ↑ astrocyte & microglia density; ↑ TNF-α and IL-6 (brain, hippocampus); neuronal degeneration with cytoplasmic shrinkage, vacuolization, necrosis and nuclear pyknosis; ↓ NeuN-positive neurons in cortex and hippocampus.	[34]

Abbreviations: M, male; F, female; U, unspecified; i.p., intraperitoneal; i.c.v., intracerebroventricular; s.c., subcutaneous; i.g., intragastric; p.o., oral; NR, not reported

## Mechanistic positioning of phytochemicals

**Fig. 3:**
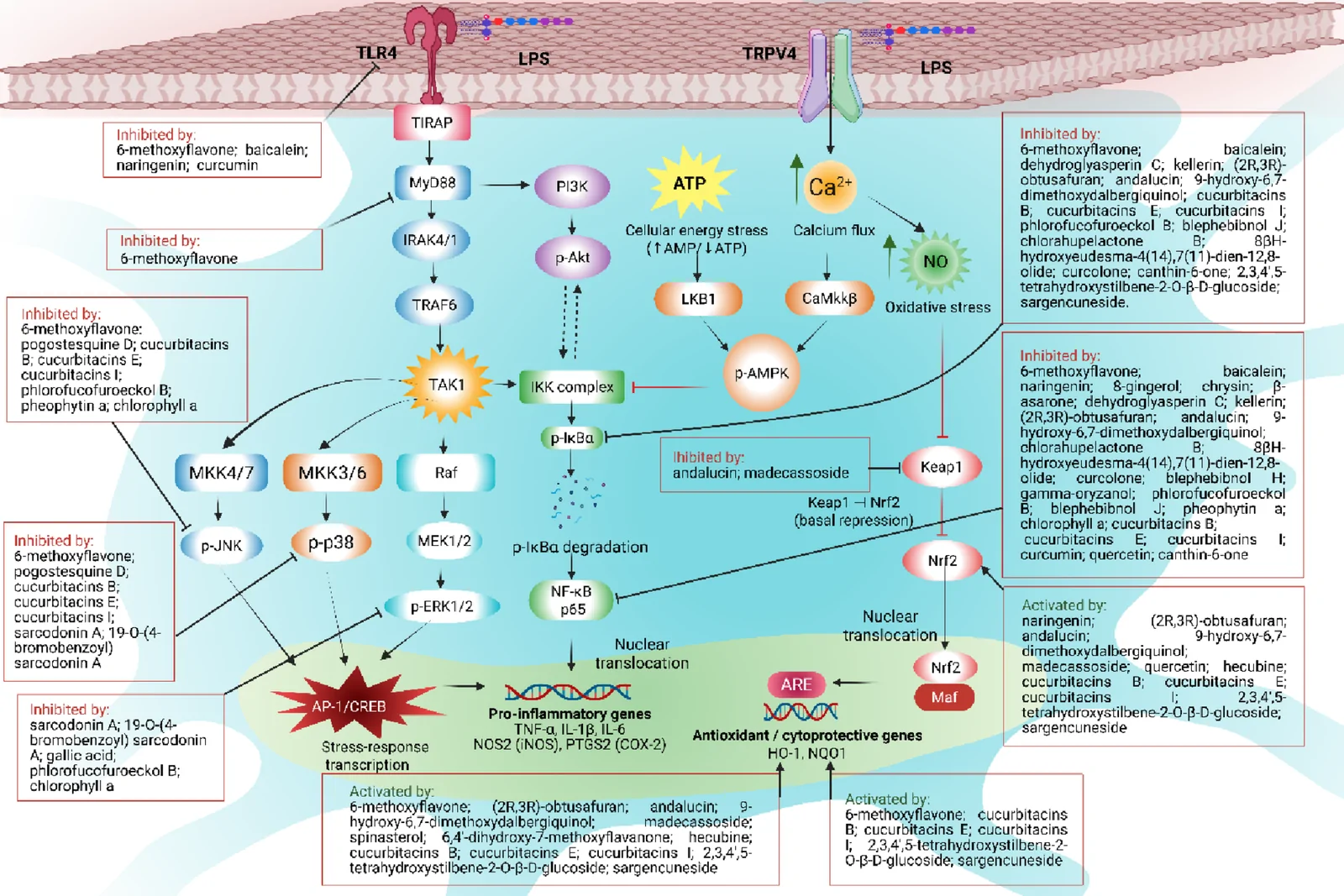
Mechanistic positioning of phytochemicals in LPS-induced neuroinflammation models. LPS engages TLR4 at the plasma membrane and signals through the TIRAP–MyD88 axis, converging via IRAK4/1 and TRAF6 on the central hub TAK1. TAK1 drives IKK–IκBα–NF-κB signalling to induce pro-inflammatory gene expression, including TNF-α, IL-1β, IL-6, NOS2/iNOS and PTGS2/COX-2, while also coordinating MAPK signalling through the MKK4/7–JNK, MKK3/6–p38 and Raf–MEK1/2–ERK1/2 modules, converging on transcription factors such as AP-1/CREB. Crosstalk with PI3K–Akt and energy-sensing pathways (LKB1/CaMKKβ–AMPK, with TRPV4-associated Ca2 + flux shown) links inflammatory activation to metabolic stress and redox regulation. Oxidative and nitrosative stress further interface with the Keap1–Nrf2 axis, in which release of Keap1-mediated repression permits Nrf2 nuclear translocation, association with small Maf proteins, binding to ARE elements and induction of cytoprotective genes, including HO-1 and NQO1. Annotated phytochemicals indicate representative compounds reported to modulate specific signalling nodes or modules in the reviewed literature, thereby extending the figure from pathway architecture to mechanistic compound positioning. Created in BioRender. Zhou, P. (2026) https://BioRender.com/ubrzdsq

From a phytochemical-screening perspective, the reviewed evidence can be stratified into levels of support. Compounds showing only NO or cytokine suppression in a single immortalised microglial model should be considered preliminary screening hits. Stronger candidates are those linking mediator suppression to viability-controlled pathway engagement, such as NF-κB/MAPK/TLR4, Nrf2/HO-1 or NLRP3 modulation. Greater preclinical priority may be given to compounds supported by multi-endpoint activity across primary or human-relevant systems and in vivo models with brain-region, glial, oxidative-stress, neuronal injury or behavioural readouts (Fig. 3).

### Pattern-recognition receptor signalling: TLR4-MyD88

TLR4–MyD88 signalling is a recurrent receptor-proximal axis used to position phytochemicals at the level of LPS recognition and early TLR4 signalling, coupling receptor-proximal modulation to downstream NF-κB and stress-kinase activation.

A similar upstream positioning was reported for 6-methoxyflavone (6-MeOF) in BV2 microglia challenged with 100 ng/mL LPS, where 6-MeOF (3-100 µM, 1 h before LPS) reduced TNF-α/IL-1β/IL-6, PGE₂, NO and ROS while improving viability, with concurrent decreases in TLR4/MyD88, p-IκB and p-NF-κB p65 and reductions in p-p38/p-JNK accompanied by increased HO-1/NQO1. In vivo, 6-MeOF pre-treatment (25–50 mg/kg, i.p., daily ×4 days) before 5 mg/kg LPS (i.p.) in C57BL/6 mice reduced Iba-1 signal in PFC and SN, consistent with reduced MyD88 expression in SN and decreased p-p38 in PFC/SN [73].

Similarly, baicalein co-treatment (45 µM for 24 h) in BV-2 microglia with 100 ng/mL LPS plus IFN-γ 20 ng/mL for 24 h, reduced TNF-α/IL-1β/IL-6 and NO, lowered CD16 and iNOS, and increased CD206/Arg-1/YM1/2 with increased TGF-β, accompanied by reduced TLR4 expression and attenuation of IκBα/p65 phosphorylation and NF-κB p65 nuclear translocation, alongside reduced p-STAT1 [48].

In vivo evidence further supports this receptor-proximal positioning. In rats receiving repeated LPS (167 µg/kg, i.p., daily ×7 days), naringenin administered (100 mg/kg, p.o., 1 h post-LPS, daily ×7) improved memory performance (Y-maze/NOR) and reduced hippocampal TNF-α, GFAP and MDA with increased hippocampal GSH, and was mechanistically associated with reduced TLR4 and NF-κB signalling alongside increased Nrf2 and reduced COX-2/ iNOS [82].

### Canonical NF-κB signalling: IκBα/p65 activation and nuclear translocation

NF-κB p65 nuclear localisation is a widely used mechanistic anchor for positioning phytochemicals relative to LPS-induced transcriptional activation in microglial systems. Across BV-2 microglia paradigms using 100 ng/mL LPS with exposures spanning 20–24 h, multiple phytochemicals reduced NO/iNOS and pro-inflammatory cytokine outputs in association with suppression of NF-κB p65 nuclear localisation, including 8-gingerol (5–20 µM, co-treated 20 h) reducing NO, TNF-α, IL-1β, IL-6 and iNOS, chrysin (1–10 µM, 30 min pretreatment; co-treated up to 24 h) reducing NO, TNF-α, IL-1β, iNOS and COX-2, and β-asarone (10–100 µM, 30–60 min pretreatment) reducing NO, iNOS, COX-2 and TNF-α/IL-1β/IL-6 [14, 68, 90]. In the same platform, dehydroglyasperin C (1–10 µM; 12 h pretreatment; co-treated up to 24 h) reduced TNF-α with downregulated p-IκBα and NF-κB p65 nuclear localisation alongside reductions in p-p38/p-ERK and increased MKP-1. Kellerin (1–10 µM; 2 h pretreatment; co-treated 24 h with 100 ng/mL LPS) reduced TNF-α, IL-6, NO, iNOS and ROS, with downregulated p-IκBα and NF-κB p65 nuclear localisation and reduced NADPH oxidase/gp91phox [54, 91].

NF-κB suppression was also reported under shorter, higher-dose LPS challenge conditions. For example, BV-2 microglia exposed to 4 µg/mL LPS for 3 h showed reduced IL-1β and TNF-α with decreased p-IκBα and p-p65 following pretreatment/co-incubation with chlorahupelactone B (1–12 µM), 8βH-hydroxyeudesma-4(14),7(11)-dien-12,8-olide (4–20 µM) or curcolone (1–8 µM) given 1 h before LPS and co-incubated during the 3 h exposure [43]. Similarly, blephebibnol H (3a) reduced TNF-α and IL-1β mRNA expression with decreased NF-κB p65 phosphorylation alongside reduced PTP1B overexpression in BV2 cells exposed to 2 µg/mL LPS, for 24 h [13].

 In vivo, gamma-oryzanol (100 mg/kg/day, oral gavage, daily for 14 days with 7 d pre-LPS + 7 d co-treatment) in BALB/c mice receiving 0.75 mg/kg/day LPS (i.p., daily for 7 days) improved memory (MWM; Y-maze), reduced Iba-1 and GFAP expressions in CA1/CA3 hippocampus and reduced hippocampal TNF-α and IL-6 levels, which was associated with attenuated hippocampal NF-κB signalling [76]. Transcript-level NF-κB modulation was also reported in BV2 microglia treated with 500 ng/mL LPS (with IFN-γ 0.5 ng/mL), where thymoquinone (12.5 µM, treated 24 h) reduced IL-6 with decreased NF-κB target genes (IL6/CFB/C3/CCL5/CXCL3) [44].

### Redox-inflammation coupling and cytoprotection: Nrf2/Keap1/HO-1

In BV2 microglia challenged with 500 ng/mL LPS for 18 h, (2R,3R)-obtusafuran (1–20 µM; 12 h pretreatment; co-treated with LPS for 18 h) reduced NO, PGE₂, TNF-α and IL-1β and decreased iNOS and COX-2 expression, alongside downregulated p-IκBα and NF-κB p65 nuclear localisation with increased Nrf2 (nuc)/HO-1 signaling [53]. In BV2 microglia challenged with 100 ng/mL LPS for 6–24 h, andalucin (5–20 µM; 1 h pretreatment; co-treated 6–24 h) significantly reduced NO, PGE₂, TNF-α/IL-6/IL-1β and iNOS/COX-2 with increased GSH, accompanied with decreased p-IκBα and NF-κB p65 nuclear localisation and increased Nrf2 (nuc)/HO-1 with Keap1 reduction [15]. A related configuration was observed for 9-hydroxy-6,7-dimethoxydalbergiquinol (HDDQ) in BV2 microglia exposed to 1 µg/mL LPS (18 h for mediators; 1 h for NF-κB readouts), where HDDQ (5–40 µM; 12 h before LPS) reduced NO, PGE₂, TNF-α/IL-1β and iNOS/COX-2, with decreased p-IκBα and NF-κB p65 nuclear localisation and increased Nrf2/HO-1 [63].

 In vivo, Nrf2/HO-1-associated reporting extended to central cytokine endpoints. For example, madecassoside (120 mg/kg, i.g., daily ×14 before LPS) in Sprague-Dawley rats challenged with 150 µg/5 µL LPS (i.c.v.) improved memory performance in the MWM and reduced TNF-α/IL-1β/IL-6 in cortex/hippocampus with increased Nrf2/HO-1 signalling, reduced Keap1 and enhanced nuclear Nrf2 [79]. In a complementary rat paradigm (167 µg/kg LPS, i.p., daily for 7 days), naringenin modulated the TLR4–NF-κB–Nrf2 axis in parallel with attenuation of hippocampal inflammatory and oxidative stress readouts, reduced GFAP, and improved memory-related behavioural performance [82].

HO-1 induction was also reported as a proximal mechanistic anchor in BV2 microglia exposed to 0.5 µg/mL LPS, where spinasterol (10–40 µM; 12 h before LPS; LPS 6–18 h) reduced NO, PGE₂, TNF-α/IL-1β and iNOS/COX-2 with increased p-ERK and HO-1 [9]. A closely aligned profile was described for 6,4′-dihydroxy-7-methoxyflavanone (DMF) in BV2 microglia exposed to 0.5 µg/mL LPS for 18 h, where DMF (10–80 µM; 12 h before LPS) reduced NO, PGE₂, TNF-α, IL-1β and iNOS with increased p-ERK and HO-1 [92].

### Stress-activated kinase cascades and mediator control: MAPK (p38/JNK/ERK1/2)

Beyond NF-κB-linked readouts, several studies explicitly incorporated MAPK phosphorylation endpoints as supporting evidence for suppressed mediator output, including pogostesquine D in BV-2 microglia exposed to 1 µg/mL LPS where pretreatment with 40–80 µM (2 h before LPS; 24 h exposure) reduced TNF-α, IL-6, iNOS and COX-2 with decreased p-NF-κB and decreased p-p38/p-JNK [93]. In primary microglia (mouse, ICR) challenged with 1 µg/mL LPS for 24 h, cucurbitacins B/E/I (0.5 µM, 1 h before LPS) reduced TNF-α/IL-1β/IL-6 and reduced iNOS/COX-2 with decreased p-IκBα and NF-κB p65 nuclear localisation and decreased p-p38/p-JNK, while also incorporating p-JAK1/p-STAT1/p-STAT3 reductions and Nrf2/HO-1/NQO-1 increases as parallel mechanistic supports [18].

MAPK reporting also extended to ERK-focused nodes across human microglia and BV-2 systems. In HMC3 microglia exposed to 100 ng/mL LPS for 24 h, sarcodonin A and 19-O-(4-bromobenzoyl) sarcodonin A (3–10 µM; 1 h pretreatment; co-treated 24 h) reduced IL-6 and COX-2 with decreased p-ERK1/2 and p-p38 alongside decreased NF-κB p65 nuclear localisation [17]. In BV-2 microglia exposed to 1 µg/mL LPS, gallic acid (170 ng/mL; co-treated 24 h) reduced IL-6 and iNOS with decreased p-ERK1/2 [94].

### Bidirectional PI3K/Akt signalling

PI3K/Akt signalling is variably used to position phytochemicals either as pathway activators linked to anti-inflammatory efficacy or as suppressed signalling modules in concert with broader inflammatory signalling inhibition, depending on the experimental paradigm. PI3K/Akt involvement was positioned with both cellular and behavioural endpoints, including sulforaphane (SFN) in primary microglia (mouse) exposed to 1 µg/mL LPS where SFN (30 µM; pretreatment duration not reported) reduced TNF-α and IL-1β and was placed within a PI3K/Akt- activation framework supported by Akt inhibition (LY294002/VIII) abrogated these effects [56]. In C57BL/6J mice receiving 100 µg/kg LPS (i.p., single injection), SFN (5 mg/kg, i.p., once daily ×5 days before LPS) reduced depression-like behaviour (forced swim test, tail suspension test) and anhedonia (sucrose preference test), with increased PI3K/Akt signalling in prefrontal cortex (PFC) and blockade of behavioural effects by Akt inhibition [56].

In BV-2 microglia, PI3K/Akt was also reported as a suppressed node in parallel with NF-κB and MAPK inhibition. Phlorofucofuroeckol B (PFF-B) in BV-2 cells exposed to 0.1 µg/mL LPS reduced NO, PGE₂, iNOS, COX-2 and TNF-α/IL-1β/IL-6 with decreased p-IκBα and NF-κB p65 nuclear localisation, decreased p-ERK/p-JNK and decreased PI3K/Akt, under a dosing range of 0–40 µM (co-treated 24 h for mediators; specific timing variants for NF-κB and kinase readouts) [95]. AKT phosphorylation suppression was also reported for blephebibnol J in BV-2 microglia exposed to 2 µg/mL LPS for 24 h, where 2.2–20 µM (2 h before LPS for WB; 4 h before LPS for NO assay) reduced NO and iNOS with decreased p-AKT and decreased p-IκBα/p-NF-κB p65 [13].

### Co-stimulation signalling in LPS + IFN-γ paradigms: JAK/STAT1

In BV-2 microglia co-stimulated with 100 ng/mL LPS plus IFN-γ 10 U/mL, both pheophytin a (0.5–2.5 µg/mL) and chlorophyll a (1–5 µg/mL) administered 1 h before co-treatment and maintained up to 24 h reduced NO and TNF-α/IL-1β/IL-6 and were associated with decreased NF-κB p65 nuclear localisation alongside attenuated JAK/STAT1 signalling, with concurrent reductions in stress kinase phosphorylation (p-JNK for pheophytin a; p-JNK/p-ERK for chlorophyll a) [10]. STAT1-linked positioning also appeared in BV-2 microglia exposed to 100 ng/mL LPS plus IFN-γ 20 ng/mL, where baicalein (45 µM, co-treated 24 h) reduced cytokines and NO and increased CD206/Arg-1/YM1/2 with increased TGF-β, in parallel with reduced p-STAT1 alongside reduced TLR4 and NF-κB p65 nuclear translocation [48].

### Signal termination and negative feedback in inflammatory signalling: MKP-1

MKP-1 induction is occasionally used as a phosphatase-mediated negative-feedback mechanism to explain coordinated attenuation of MAPK and NF-κB signalling in LPS-challenged microglia. For example, dehydroglyasperin C (DGC) in BV-2 microglia exposed to 100 ng/mL LPS was positioned with MKP-1 induction as a mechanistic feature, where DGC (1–10 µM; 12 h pretreatment; co-treated with LPS up to 24 h) reduced TNF-α with decreased p-IκBα and reduced NF-κB p65 nuclear localisation, alongside reductions in p-p38/p-ERK and increased MKP-1 [91].

### Immunometabolic control of inflammatory transcription: PKM2/HIF-1α (PIN1; nuclear PKM2)

Immunometabolic positioning is occasionally used to connect LPS-driven inflammatory outputs to glycolysis-linked transcriptional control, with PKM2–HIF-1α features providing a mechanistic anchor in some microglial paradigms. Metabolic-node positioning was reported for coptisine in BV-2 microglia exposed to 250 ng/mL LPS for 24 h, where coptisine (30 µM; 1 h before LPS) reduced NO, TNF-α, IL-1β and iNOS with decreased p-PKM2 (Y105/S37), decreased PIN1, reduced nuclear PKM2 and decreased HIF-1α [41] In vivo, C57BL/6 mice receiving 0.83 mg/kg LPS (i.p.) attenuated anxiety-like behaviour (OFT/EPM) and increased NeuN with reduced Iba-1 and reduced IL-1β and TNF-α following oral coptisine (15–60 mg/kg/day, daily ×7 before LPS), with mechanistic reporting framed as PKM2 reduction [41].

### Microglial receptor-state modulation and phagocytic programming: TREM2

TREM2 upregulation is used in a subset of studies to position phytochemicals within microglial receptor-state modulation, linking innate sensing to phagocytic function and downstream inflammatory outputs. Microglial receptor repositioning was reported for curcumin in BV2 microglia exposed to 1 µg/mL LPS for 24 h, where curcumin (1–10 µM; 2 h pretreatment then co-treated 24 h) reduced NO, IL-1β, IL-6 and iNOS while increasing phagocytosis, associated with reduced TLR4 expression and decreased p-IκBα/NF-κB p65 nuclear localisation with increased TREM2 [47].

In zebrafish larvae (AB wild-type) receiving LPS by brain microinjection (2.5 mg/mL), hecubine (6–25 µM; immersion 24 h before LPS) increased motor activity and reduced NO and ROS with reduced Iba-1 and reduced TNF-α/IL-1β/IL-6, accompanied by increased TREM2 with increased Nrf2/HO-1 [88].

### Mitophagy-inflammasome coupling and pyroptotic effector control: PINK1/Parkin

PINK1/Parkin-linked mitochondrial quality control is used in selected LPS paradigms to position phytochemicals at the interface between inflammasome activation, pyroptotic signalling and redox stress. Quercetin provides a well-characterised example across both in vitro and in vivo systems. In BV-2 microglia exposed to 100 ng/mL LPS for 24 h, quercetin (30–60 µM; 1 h before LPS) reduced TNF-α/IL-1β/IL-6, iNOS and ROS with decreased p-IKKβ and reduced NF-κB p65 nuclear localisation alongside increased nuclear Nrf2. Under inflammasome-activation conditions (100 ng/mL LPS for 24 h plus ATP 5 mM for 30 min), quercetin (30 µM; 1 h before LPS) reduced IL-1β release and pyroptosis (YO-PRO-1 uptake), with decreases in NLRP3, cleaved caspase-1 and GSDMD-N and increased LC3-II, decreased p62 and increases in PINK1/Parkin alongside reduced mitochondrial ROS [12].

 In vivo, quercetin (30–60 mg/kg, i.p., daily; 3 d before LPS and during 5 d LPS) in C57BL/6 male mice exposed to 1 mg/kg LPS (i.p., daily ×5) reduced Iba-1 (hippocampus/cortex) and CD68 and reduced immobility time (TST/FST), with mechanistic evidence including reduced NLRP3, reduced active caspase-1 and reduced IL-1β alongside increased PINK1 [12]. A related behavioural-neuroimmune association was reported in rats (male, 17–18 weeks; strain not reported) receiving 0.85 mg/kg LPS (i.p., single), where quercetin (50 mg/kg, p.o., daily ×7; last dose 60 min before LPS) reduced TNF-α in hippocampus/PFC and reduced IL-6 in hippocampus with reduced Iba-1 and iNOS in PFC/hippocampus CA1 and reduced depression-like behaviour, in association with reduced NF-κB and NLRP3 [81]. In adult zebrafish (470–530 mg) receiving 1 mg/kg LPS (i.p., single injection), quercetin (50–100 mg/kg, i.p., post-LPS for 7 days) reduced TNF-α, IL-1β, MDA and NO with increased GSH, although pathway-level mechanism was not specified [71].

### Inflammasome effector programme and cytokine maturation: NLRP3/caspase-1/IL-1β

Inflammasome-linked endpoints (NLRP3/caspase-1/mature IL-1β) are variably reported but provide a more direct mechanistic layer in some glial LPS paradigms [96]. In C8-D1A astrocytes exposed to 1 µg/mL LPS for 18 h, canthin-6-one (6.25-25 µM; 6 h pretreatment before LPS; maintained during 18 h exposure) reduced NO, TNF-α, IL-1β and IL-6 and ROS, with concurrent reductions in p-IκBα and NF-κB p65 nuclear localisation and decreases in STAT3 alongside increased Nrf2/HO-1 and reductions in NLRP3/caspase-1/IL-1β [27]. In BV-2 microglia exposed to 500 ng/mL LPS, evolecoumarin C (1-100 µM; co-treated; duration not reported) reduced NO, IL-1β and IL-6 with reduced NLRP3 expression [40].

## Translational interpretation and limitations of LPS-induced neuroinflammation models

This review has several methodological limitations. It was designed as a narrative review informed by structured searching rather than a formal systematic review; therefore, no risk-of-bias or study-quality assessment was performed. In addition, heterogeneity in LPS preparation, model systems, dosing schedules, sampling times and endpoint reporting limited direct comparison across studies.

### What the LPS platform can and cannot support

LPS-induced neuroinflammation is best interpreted as a controlled innate immune challenge rather than a direct surrogate for complex chronic CNS diseases [3, 6]. Its principal experimental value lies in its capacity to activate TLR4-linked inflammatory signalling and generate reproducible mediator and glial activation outputs, including TNF-α, IL-1β, IL-6, iNOS/NO, ROS and glial activation markers [12, 15].

Acute LPS paradigms generally involve a single or short-term exposure and are useful for resolving rapid TLR4-dependent signalling, cytokine production, oxidative stress and glial activation. In contrast, chronic neuroinflammation models use repeated or prolonged stimulation to capture sustained glial activation, impaired inflammatory resolution, cumulative neuronal dysfunction and longer-term tissue remodelling. Acute LPS models provide greater experimental control and reproducibility, whereas chronic paradigms offer stronger relevance to persistent inflammatory states but introduce greater biological and methodological complexity [41, 54].

### Sources of variability and reproducibility concerns

A major limitation of LPS-induced neuroinflammation research is inconsistent reporting of LPS preparation, including bacterial source/serotype, supplier, catalogue details, purification grade and dose, as summarised in Tables 1 and 2. For example, in vitro studies use diverse LPS preparations and concentrations ranging from ng/mL to µg/mL, whereas in vivo studies vary in species, route of administration, dose, dosing schedule and sampling time. These variables can alter TLR4 activation potency, signalling kinetics and downstream inflammatory outputs, and may also introduce additional pattern-recognition signals. Incomplete reporting of exposure duration, route, serum conditions and sampling time therefore reduces reproducibility, weakens mechanistic attribution and makes direct inter-study comparison of phytochemical effects difficult.

Timing is another major determinant of interpretation because early signalling events and later inflammatory phenotypes reflect distinct biological phases of the LPS response [9, 10]. Early readouts such as IκBα phosphorylation, NF-κB p65 nuclear translocation and MAPK phosphorylation are usually most informative within minutes to early hours after LPS exposure. Later readouts such as iNOS/NO, COX-2/PGE2, cytokine release, ROS accumulation, cell viability, microglial morphology and glial activation markers are more appropriately interpreted as transcriptional, protein-level or phenotype-level inflammatory outputs [15, 54]. Misalignment between sampling time and endpoint biology can therefore weaken mechanistic attribution and reduce comparability across studies.

 In vivo studies are subject to additional variability arising from species, strain, sex, age, route of LPS administration, dosing schedule, brain region sampled and the timing of LPS exposure, behavioural testing and tissue collection [70, 77, 81]. Systemic LPS paradigms engage peripheral immune signalling and sickness-like physiology and should therefore not be interpreted as models of isolated CNS neuroinflammation. Attribution to CNS inflammation is strengthened by brain-region-specific inflammatory, glial or neuronal readouts, whereas behavioural changes may partly reflect systemic immune activation. Central routes such as intracerebroventricular administration more directly evoke CNS inflammation but may not fully capture peripheral-to-central inflammatory communication. Consequently, in vivo phytochemical effects should be interpreted in relation to administration route, exposure kinetics, tissue distribution, behavioural context and region-specific neuroinflammatory readouts [69, 76].

### *In vitro–in vivo* coherence and hierarchy of claims

 In vitro models are useful for first-pass screening, dose–time optimization and pathway mapping, but their translational value depends on the biological relevance of the model and the appropriateness of the selected endpoints [97, 98]. BV-2 and N9 microglial cells provide scalable and reproducible systems for NO/iNOS, cytokine and NF-κB/MAPK-linked readouts, but immortalized microglial lines do not fully reproduce primary or adult human microglial states. Primary microglia cultures provide greater physiological relevance by allowing assessment of activation markers, morphology, phagocytosis and broader inflammatory phenotypes, although isolation procedures and culture conditions can introduce variability [17, 18]. Human microglia-like and myeloid platforms, including HMC3, CHME3 and THP-1-derived systems, can strengthen translational alignment but should be interpreted with attention to cell identity, differentiation state and reporter-specific limitations [24–26].

 In vivo models incorporate systemic physiology, brain-region context, glial activation and behavioural relevance, but they also introduce pharmacokinetic and biological complexity that cannot be inferred from in vitro activity alone [74]. Across animal studies, translationally useful readouts commonly combine cytokines, iNOS/NO-related mediators, oxidative-stress markers, Iba-1/AIF-1, GFAP, neuronal injury indices and behavioural outcomes [78, 81]. Zebrafish models provide an intermediate in vivo platform for inflammation-linked locomotor, NO/ROS and cytokine readouts, but their dosing routes and developmental context require careful interpretation relative to rodent or mammalian CNS models [71, 73].

To stratify heterogeneous studies, we propose a pragmatic four-level hierarchy applied to the strength of the supported claim rather than to model type alone. Level 1 (preliminary) comprises a viability-controlled change in inflammatory markers in a single model. Level 2 (mechanistic) requires concordant early-signalling and downstream mediator changes, supported by replication or dose–response evidence. Level 3 (cross-model) requires multi-endpoint confirmation in an independent, primary, human-relevant or multicellular platform. Level 4 (preclinical) requires coherent in vitro and in vivo findings, including brain inflammatory or glial endpoints together with functional outcomes. Behaviour-only animal findings do not automatically constitute Level 4 evidence, and studies sharing the same methodological limitations do not increase confidence. These criteria reflect recurring study-design and endpoint patterns in Tables 1 and 2; their organisation into confidence levels represents pragmatic author-derived guidance rather than a formal risk-of-bias or GRADE assessment.

Overall, LPS-induced neuroinflammation models may offer greater translational value when they are used as kinetics-aware pharmacological screening systems rather than disease-equivalent models. Standardized reporting of LPS source, serotype, purity, dose, timing, route, biological context, phytochemical exposure and endpoint rationale is essential to improve reproducibility and cross-study comparability. The confidence hierarchy should therefore be applied alongside the model-specific guidance presented in Table 3.

**Table 3:** Kinetics-informed hierarchy for selecting LPS-induced neuroinflammation models and interpreting phytochemical evidence

Evidence level and primary research objective	Commonly used models and applications	LPS design and kinetically aligned endpoints	Validation considerations and indicative progression	Justified claim and interpretive boundary
Level 1: Preliminary First-pass phytochemical screening	Immortalised microglia, such as BV-2 or N9, for rapid screening and dose–time optimisation	Report LPS source/serotype, purity, supplier, concentration and exposure duration. Prioritise late outputs such as NO/iNOS, cytokines, COX-2/PGE₂ and ROS, together with viability/cytotoxicity	Vehicle, LPS-only and compound-only controls; expected LPS responsiveness; dose-related activity without nonspecific cytotoxicity. Proceed: reproducible activity without toxicity. Refine: weak or time-dependent response. Stop: inadequate LPS responsiveness or nonspecific toxicity	Preliminary anti-inflammatory activity limited to the tested model, exposure conditions and endpoints
Level 2: Mechanistic Temporal/kinetic characterisation or mechanistic investigation	Immortalised microglia for kinetic mapping; primary microglia for physiological and mechanistic confirmation	Early (≤ 2 h): NF-κB, MAPK, PI3K/Akt, STAT/IRF and TLR4–MyD88. Intermediate (2–8 h): iNOS/cytokine transcription, NLRP3 priming and Nrf2/ARE–HO-1. Late (12–60 h): mediators, morphology, phagocytosis and viability	Level 1 criteria plus concordant proximal signalling and downstream responses; independent replication or dose–response support; causal perturbation where feasible. Proceed: replicated, time-aligned multi-endpoint activity. Refine: discordant endpoints or unsuitable sampling. Stop: non-reproducible activity or toxicity	Supported pathway modulation. Causality is justified only when directly tested
Level 3: Cross-model Human-relevance or multicellular translational validation	Primary microglia; human microglia-like or myeloid systems; neuronal or mixed-cell cultures	Apply model appropriate LPS exposure and sampling. Assess cytokines, Iba1/CD68, morphology, phagocytosis, neuronal viability, apoptosis and cell–cell interactions at appropriate intervals	Level 2 criteria plus concordant multi-endpoint effects in an independent, biologically distinct platform; cell identity and differentiation appropriately controlled. Proceed: convergent effects across biologically distinct models, together with acceptable exposure and safety feasibility. Refine: optimise model state, exposure or endpoints. Stop: effects remain model-specific or non-reproducible	Cross-model biological coherence and increased human or multicellular relevance; systemic exposure and in vivo relevance remain unconfirmed
Level 4: Preclinical System-level or whole-organism validation	Zebrafish or rodent models integrated with supporting in vitro evidence	Report species, developmental stage or age, sex, LPS dose, route, schedule and sampling interval. Assess region-specific cytokines, Iba1/GFAP, oxidative stress, neuronal injury and functional or behavioural outcomes	Level 3 criteria plus tolerability or exposure evidence, target engagement where feasible, tissue inflammatory or glial endpoints and functional outcomes. Proceed: convergent molecular, tissue and functional evidence. Refine: inadequate exposure, timing or model responsiveness. Stop: nonspecific toxicity or incoherent cross-model findings	Preclinical in vivo support, but not disease-modifying, clinical or therapeutic proof

### Criteria for progression from in vitro to in vivo studies

Within this author-derived hierarchy, Level 3 is proposed as a pragmatic decision point for considering progression to in vivo testing because it indicates that activity has been confirmed in a biologically distinct platform rather than remaining confined to a single in vitro model. It is not an empirically validated or universally applicable threshold, and progression should remain context-dependent according to the proposed mechanism of action, pharmacokinetic and exposure characteristics, therapeutic objective, biological question or disease context, availability of suitable in vitro or multicellular models, and safety and formulation feasibility. Factors that may support progression include: (i) reproducible concentration-dependent effects at non-cytotoxic concentrations; (ii) modulation of at least two complementary, kinetics-aligned endpoints, including a proximal signalling event and a downstream inflammatory or functional outcome; (iii) confirmation in a second biologically relevant platform, preferably a primary, human-relevant or multicellular system; and (iv) sufficient solubility, stability, formulation and preliminary toxicity information to justify the proposed dose and administration route. Causal perturbation should be sought where feasible when progression is justified on the basis of a specific molecular target. BBB relevance should be evaluated when the proposed mechanism requires CNS exposure; experimental or pharmacokinetic evidence is preferred, whereas computational prediction should remain supportive. Direct BBB penetration is not essential when a peripheral-to-central immunomodulatory mechanism is explicitly proposed. By contrast, marker suppression in a single cell line without cytotoxicity exclusion, reproducibility or exposure feasibility would generally provide insufficient support for progression within this framework. Subsequent in vivo studies should assess exposure or tolerability, brain-region inflammatory or glial endpoints, and functional outcomes aligned with LPS kinetics.

## Future directions

Future studies should move from single-timepoint phytochemical screening toward kinetics-aware experimental designs that distinguish early LPS sensing from later mediator production and phenotype-level injury. This distinction is pharmacologically important because suppression of a late mediator at 24 h does not necessarily demonstrate direct inhibition of early TLR4 signalling; it may instead reflect altered transcription, redox tone, altered viability or delayed feedback regulation. Inflammasome-focused studies should also distinguish LPS priming from secondary activation signals such as ATP or nigericin, enabling more precise attribution of phytochemical effects to priming, inflammasome assembly, caspase-1 activation, GSDMD cleavage or mature IL-1β release.

Future studies should increasingly incorporate human-relevant and multicellular platforms. Human iPSC-derived microglia are emerging tools because they preserve human genetic background and can model donor-specific inflammatory responses. Microglia-containing organoids provide a three-dimensional system to study longer-term glia–neuron interactions, while microfluidic BBB/neurovascular platforms allow assessment of barrier function and endothelial–glial communication. Multicellular co-cultures further integrate microglia, astrocytes, neurons and endothelial cells, better reflecting CNS inflammatory complexity than monoculture systems. These approaches can improve translational relevance beyond immortalised microglial lines and acute LPS exposure.

Publication bias and selective reporting may affect interpretation because positive phytochemical findings may be preferentially published over neutral, negative or toxicity results. Testing multiple compounds, concentrations and endpoints may also overstate consistency across LPS models. As this narrative review did not assess unpublished evidence or small-study effects, the extent of bias cannot be quantified. Prespecified endpoints and reporting of null and adverse findings would strengthen confidence.

## Conclusion

LPS-induced neuroinflammation remains a valuable experimental framework for interrogating innate immune activation in the CNS, but its utility in phytochemical research depends on disciplined interpretation of what the model can and cannot support. Across cellular and animal systems, LPS paradigms reproducibly engage a reproducible inflammatory architecture encompassing cytokine induction, iNOS/NO signalling, oxidative-stress coupling, glial activation and a restricted set of commonly mapped pathways. This consistency underpins the platform’s practical value for mechanistic triage and comparative pharmacology.

At the same time, translational interpretation is constrained by the frequent use of immortalised cell lines, acute LPS paradigms and heterogeneity in LPS source, dose, timing and endpoints. These models are valuable for controlled inflammatory screening, but they do not fully recapitulate primary human microglial biology, multicellular CNS interactions or chronic neurodegenerative pathology. Therefore, positive findings should be interpreted as evidence of pathway modulation under defined inflammatory challenge, rather than direct proof of disease-modifying efficacy.

Future progress will require stricter stimulus definition, kinetics-aligned sampling, viability-aware and state-resolving readouts, and validation across more informative human-relevant and cross-species model systems, so that phytochemical leads are advanced with greater mechanistic resolution, comparability and translational discipline, without prematurely overstating their relevance to complex disease biology.

Overall, the field now requires a shift from descriptive anti-inflammatory screening toward better standardised, mechanism-informed and kinetics-aware study designs. Priority should be given to rigorous LPS reporting, endpoint timing matched to inflammatory biology, validation in primary or human-relevant multicellular systems, and cautious interpretation of claims according to the strength of cross-model evidence.

## Supplementary Information

Below is the link to the electronic supplementary material.

Supplementary Material 1

## Data Availability

No datasets were generated or analysed during the current study.

## Abbreviations

Aβ: Amyloid-β
AD: Alzheimer’s disease
AMPK: AMP-activated protein kinase
AP-1: Activator protein 1
ARE: Antioxidant response element
BBB: Blood–brain barrier
CNS: Central nervous system
COX-2: Cyclooxygenase-2
ECAR: Extracellular acidification rate
EdU: 5-ethynyl-2′-deoxyuridine
ERK: Extracellular signal-regulated kinase
GFAP: Glial fibrillary acidic protein
GSDMD-N: N-terminal gasdermin D
HO-1: Haem oxygenase 1
IBA1: Ionized calcium-binding adapter molecule 1
ICV: Intracerebroventricular
IFN-γ: Interferon-γ
IKK: IκB kinase
IL: Interleukin
iNOS: Inducible nitric oxide synthase
IRF3: Interferon regulatory factor 3
JAK: Janus kinase
JNK: c-Jun N-terminal kinase
LC3-II: Microtubule-associated protein 1 light chain 3-II
LPS: Lipopolysaccharide
MAPK: Mitogen-activated protein kinase
MKP-1: Mitogen-activated protein kinase phosphatase 1
mtROS: Mitochondrial reactive oxygen species
MyD88: Myeloid differentiation primary response 88
NF-κB: Nuclear factor-κB
NLRP3: NLR family pyrin domain containing 3
NO: Nitric oxide
NQO1: NAD(P)H quinone dehydrogenase 1
Nrf2: Nuclear factor erythroid 2-related factor 2
OCR: Oxygen consumption rate
PD: Parkinson’s disease
PGE2: Prostaglandin E2
PI3K: Phosphoinositide 3-kinase
PINK1: PTEN-induced kinase 1
PK/PD: Pharmacokinetic/pharmacodynamic
PKM2: Pyruvate kinase M2
ROS: Reactive oxygen species
SAE: Sepsis-associated encephalopathy
STAT1: Signal transducer and activator of transcription 1
STING: Stimulator of interferon genes
TBK1: TANK-binding kinase 1
TLR4: Toll-like receptor 4
TNF-α: Tumour necrosis factor-α
TREM2: Triggering receptor expressed on myeloid cells 2
TRIF: TIR-domain-containing adapter-inducing interferon-β
