# Supplementary material for: Kinetics and mechanisms in lipopolysaccharide-induced neuroinflammation: a framework for phytochemicals screening
Source: Mol Biol Rep. 2026 Oct 8;53(1):1677. doi: 10.1007/s11033-026-12817-4 (PMC13649827; doi:10.1007/s11033-026-12817-4)
Supplement: Supplementary file 1 [file 11033_2026_12817_MOESM1_ESM.docx]

**Table S1. Choosing cellular platforms for LPS-induced neuroinflammation screening.** Representative cell lines are summarised with pragmatic strengths, limitations and best-fit applications to inform model selection and interpretation; strengths/limitations are synthesised from comparative and methodological literature on microglial culture systems and human immortalised microglia lines and from representative use of THP-1-XBlue™-MD2-CD14 reporter monocytes for TLR4 pathway benchmarking.

| Cell line (species) | Strengths | Limitations | Best-fit use |
| --- | --- | --- | --- |
| Primary microglia (mouse) | Higher physiological fidelity; functional phenotypes (phagocytosis, morphology, CD68) | Isolation/culture can prime/activate cells; batch variability; lower throughput | Mechanistic validation; phenotype-rich confirmation of hits; benchmark against immortalised lines |
| Primary microglia (rat) | Cross-species confirmation in primary cells; useful robustness check | Prep-dependent variability; lower throughput | Replication across species/platforms; validation of conserved directionality |
| BV-2 / BV2 microglia (mouse) | High throughput; robust mediator + signalling readouts; broad community precedent | Immortalised phenotype; passage-dependent drift; not homeostatic adult microglia | Primary screening; dose–time optimisation; multi-endpoint panels (cytokines, NO/iNOS, ROS, NF-κB/MAPK) |
| N9 microglia (mouse) | Alternative murine microglial background to test robustness | Immortalised; response profile can differ from BV-2 and primary microglia | Replication/robustness testing; NO/iNOS-anchored assays |
| HMC3/CHME3 microglia-like cells (human; reported under multiple names/aliases in the literature) | Human-derived, scalable platform; supports cytokine/ROS and pathway readouts | Nomenclature/identity varies across sources; phenotype can deviate from canonical microglial marker profiles—interpret as microglia-like with caution | Directionality check in a human microglia-like system; pathway-level benchmarking when authentication is confirmed |
| C8-D1A astrocytes (mouse) | Homogeneous astrocyte platform; robust cytokine/ROS outputs; useful for astroglial signalling | Not microglia; astrocyte inflammatory programmes differ from microglial innate responses | Astroglial contribution to inflammatory tone; ROS/Ca²⁺-linked inflammatory signalling |
| C6 glioma/astroglioma (rat) | Convenient glia-like platform; strong, reproducible signal | Tumour-derived glioma biology; limited physiological fidelity to primary astrocytes | Rapid glial screening; directionality cross-check of compound effects |
| Primary cortical glial cells (rat; mixed glia) | Captures glial crosstalk; allows concurrent GFAP + Iba1 readouts | Composition varies; attribution to one cell type is limited; microglia fraction/reactivity can disproportionately drive outputs | Network-level glial activation validation; “glial unit” confirmation |
| THP-1-derived macrophages (human) | Strong innate responsiveness (protocol-dependent); scalable comparator; robust cytokine outputs | Peripheral myeloid biology ≠ microglia; differentiation protocol strongly shapes phenotype | Benchmark anti-inflammatory potency; innate signalling comparator |
| THP-1-XBlue™-MD2-CD14 monocytes (human; reporter) | Quantitative NF-κB/AP-1 SEAP reporter; high-throughput; stimulus-defined | Reporter activity ≠ phenotype-level inflammation; not CNS-resident | Rapid ranking of TLR4-axis modulation (pathway-level) |
| HT22 hippocampal neurons (mouse) | Downstream neuronal stress/viability/apoptosis endpoints; links inflammation → neuronal injury | Direct LPS responsiveness is variable and often dose-dependent; best interpreted as neuronal injury/stress rather than innate immune inflammation | Secondary validation: neuronal vulnerability and stress signalling under inflammatory challenge |
| SH-SY5Y neuroblastoma (human) | Human neuronal context; scalable injury endpoints | Cancer-derived; differentiation-state variability; inflammatory responsiveness depends on conditioning | Downstream neuronal endpoints; translational directionality (injury/stress modules) |
| dPC12 pheochromocytoma cells (rat) | Strong oxidative/mitochondrial phenotypes; convenient for ROS/mitochondrial assays | Tumour-derived neuron-like model; not CNS neuron; high-dose paradigms can be harsh | ROS/mitochondria-centric injury modules; stress-pathway interrogation |

A. **Rodent regional sampling emphasis**

B. **Challenge architecture across species and routes**

**Fig. S1. Design-to-biology map of *in vivo* LPS neuroinflammation models**. **A**, Rodent regional sampling most commonly emphasizes the hippocampus and cortex, with additional sampling of the prefrontal cortex, striatum and amygdala; hippocampal are frequently resolved in region-specific analyses. **B**, *In vivo* LPS paradigms are organized by route and timing, with rodent studies dominated by systemic intraperitoneal single-dose and repeated-dose designs, complemented by central delivery approaches, while zebrafish models use embryo immersion or larval brain injection. Endpoint collection spans acute, early post-acute, multi-day and delayed windows, illustrating how challenge architecture shapes anatomical focus, phenotypic depth and mechanistic interpretability. Created in BioRender. Zhou, P. (2026) <https://BioRender.com/x2yrwfp>.
